# Comparative proteomics of three Chinese potato cultivars to improve understanding of potato molecular response to late blight disease

**DOI:** 10.1186/s12864-020-07286-3

**Published:** 2020-12-09

**Authors:** Chunfang Xiao, Mengling Huang, Jianhua Gao, Zhen Wang, Denghong Zhang, Yuanxue Zhang, Lei Yan, Xiao Yu, Bo Li, Yanfen Shen

**Affiliations:** 1 State Key Laboratory of Agricultural Microbiology and Hubei Key Laboratory of Plant Pathology, College of Plant Science and Technology, Huazhong Agricultural University, Wuhan, 430070 Hubei China; 2 Southern Potato Research Center of China, Enshi, 445000 Hubei China; 3 Enshi Tujia and Miao Autonomous Prefecture Academy of Agricultural Sciences, Enshi, 445000 Hubei China

**Keywords:** Comparative proteomics, Potato cultivars, *Phytophthora infestans*, Late blight disease, Hypersensitive response, Susceptible, Tolerance, Resistant

## Abstract

**Background:**

Late blight disease (LBD) caused by the pathogen *Phytophthora infestans* (PI), is the most devastating disease limiting potato (*Solanum tuberosum*) production globally. Currently, this disease pathogen is re-emerging and appearing in new areas at a very high intensity. A better understanding of the natural defense mechanisms against PI in different potato cultivars especially at the protein level is still lacking. Therefore, to elucidate potato proteome response to PI, we investigated changes in the proteome and leaf morphology of three potato cultivars, namely; Favorita (FA), Mira (MA), and E-malingshu N0.14 (E14) infected with PI by using the iTRAQ-based quantitative proteomics analysis.

**Results:**

A total of 3306 proteins were found in the three potato genotypes, and 2044 proteins were quantified. Cluster analysis revealed MA and E14 clustered together separately from FA. The protein profile and related functions revealed that the cultivars shared a typical hypersensitive response to PI, including induction of elicitors, oxidative burst, and suppression of photosynthesis in the potato leaves. Meanwhile, MA and E14 deployed additional specific response mechanism different from FA, involving high induction of protease inhibitors, serine/threonine kinases, terpenoid, hormone signaling, and transport, which contributed to MA tolerance of LBD. Furthermore, inductions of pathogenesis-related proteins, LRR receptor-like kinases, mitogen-activated protein kinase, WRKY transcription factors, jasmonic acid, and phenolic compounds mediate E14 resistance against LBD. These proteins were confirmed at the transcription level by a quantitative polymerase chain reaction and at the translation level by western-blot.

**Conclusions:**

We found several proteins that were differentially abundant among the cultivars, that includes common and cultivar specific proteins which highlighted similarities and significant differences between FA, MA, and E14 in terms of their defense response to PI. Here the specific accumulation of mitogen-activated protein kinase, Serine/threonine kinases, WRKY transcription played a positive role in E14 immunity against PI. The candidate proteins identified reported in this study will form the basis of future studies and may improve our understanding of the molecular mechanisms of late blight disease resistance in potato.

**Supplementary Information:**

The online version contains supplementary material available at 10.1186/s12864-020-07286-3.

## Background

*Phytophthora infestans* (PI), the causative agent of late blight disease (LBD) of the family Solanaceae, is re-emerging and appearing in new areas at very high intensity [1]. When control fails, LBD epidemy damage foliage and tubers, which can lead to total crop failure, especially in potato (*Solanum tuberosum*) [1, 2]. LBD was responsible for potato famine in Europe in the nineteenth century which led to several deaths [3], and to date, LBD remains a global food security threat with an estimated cost in billions of dollars in control measures and crop losses [4, 5]. The predicted rise in global temperature could upsurge LBD incidence, particularly in humid areas [6], and may lead to the emergence of new aggressive PI strains, and worsen the challenges already facing potato industries around the world.

Host genetic resistance is the most sustainable mechanism to combat PI, and some members of the Solanaceae family are known to maintain a range of locus diversity for LBD resistance [7]. However, evidence of partial or complete breakdown of some resistance (R) loci has emerged [8, 9], which underscores the need to explore additional sources of LBD resistance within potato germplasm to understand the molecular mechanism underpinnings different types of potato resistance to LBD. Such information will be useful for developing breeding strategies for combatting LBD.

Generally, host defense and immunity against pathogenic attack initiate with the recognition of highly conserved pathogen-associated molecular patterns (PAMPs) by the cell surface pattern-recognition receptors (PRRs), which trigger host immunity (PAMP-triggered immunity-PTI) [10]. However, our knowledge of PRRs in potato is limited. PI colonizes host cells by suppressing basal immunity with an array of effector proteins, leading to effector-triggered susceptibility (ETS) [4, 5, 11]. Through evolution, host plants have evolved dominant R genes to counter ETS [5, 12]. Most R genes code for proteins with N-terminal nucleotide-binding site (NBS) and C-terminal leucine-rich repeat (LRR) that recognize pathogen effectors, and establish effector-triggered immunity (ETI) [12, 13]. However, to date, a comprehensive understanding of potato proteins involved in ETI and associated biological processes and molecular mechanisms that result in hypersensitive-response (HR-phenotype)-related programmed cell death (PCD) and overall immunity against PI is still lacking [14]. Through transcriptomic studies [12, 15, 16] the transcriptional response of potato to PI effectors are well understood, less understood, however, is the PI effector-induced changes in potato at the proteome level. This has remained a challenge because (1) mRNA does not always provide information on protein abundance across disease conditions [17]. (2). Protein synthesis can be further regulated at the translational and post-translational level, a phenomenon common in plant responses to stress, (3) Proteins ultimately control biological processes. Therefore, the proteomic landscape provides a holistic view of potato response to PI invasion.

Label-free and labeled quantitative proteomics has become a favorite tool to quantify global changes in protein abundance during plant and pathogen interactions, and to identify associated biological and molecular processes including candidate proteins underlying susceptible, tolerance, or resistance against pathogens [18, 19]. For example, methods like isobaric tags for relative and absolute quantification (iTRAQ) and tandem mass tags (TMT) are routinely used by different platforms because they are compatible with samples from multiple sources [20]. Its potential has been demonstrated in many crop species, including potato response to PI [4], potato cell wall proteins associated with PI pathogenicity [21], and protein profiling of potato leaf tissues [19].

This study reports the response of three Chinese potato varieties: Favorita (FA), Mira (MA), and E-malingshu N0.14 (E14) during potato foliage-PI interactions and revealed potato proteins, biological and metabolic functions target by PI using a combination of ITRAQ-based quantitative proteomics, western blot analysis, and quantitative real-time polymerase chain reaction (qPCR). We found that after infection of potato leaves with PI, the FA plants exhibited a gross morphology of leaves usually observed in cultivars susceptible to PI. MA exhibited similar to the cultivars tolerant to PI, while the phenotype of E14 was immune to *P. infestans*.

## Results

### Morphological response of different potato cultivars subjected to *P. infestans* infection (PI)

**Fig. 1:**
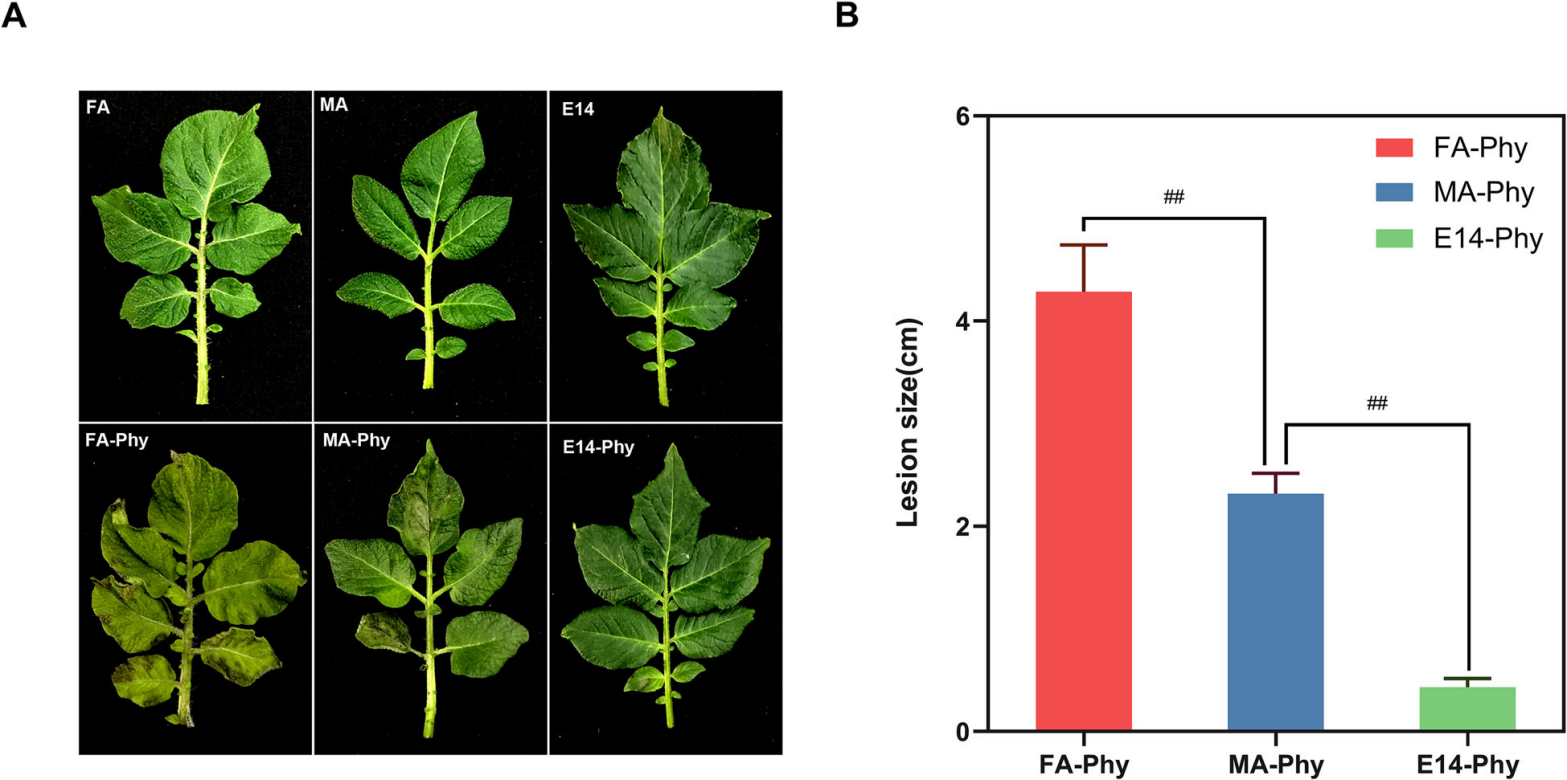
Morphological analysis of the effect of PI treatment on FA, MA and E14, and control plants. A. Morphological observation of leaves of control plants and FA-Phy, MA-Phy and E14-Phy five days after PI inoculation. B. Statistical analysis of leaf lesion diameter of FA-Phy, MA-Phy and E14-Phy 5 days after inoculation. Three replicates were used for each treatment in these tests. Bars represent the standard deviation of three replicates. Statistical significance was analyzed using Student’s t-test. The asterisk indicates the significant difference (* *p* < 0.05)

The Favorita (FA), Mira (MA), and E-malingshu NO. 14 (E14) cultivars were chosen for this study based on their frequently use as elite parents in potato breeding programmes across China [22, 23]. To examine the morphological responses of each potato cultivars to LBD pathogen PI, we scored potato leaves for disease severity at 5 dpi based on hypersensitive reaction (HR) or expanding lesion size [24]. Figure 1a shows the leaf phenotype of FA, MA, and E14 infected and control plants. The difference in disease severity indicates that FA-Phy had a higher degree of wilting and disease lesions compared to FA control, MA-Phy, and E14-Phy (Fig. 1b). In contrast, leaves of MA-Phy plants with PI had fewer signs of HR lesions compared to FA-Phy plants, but the severity of HR lesions was significantly different compared to MA control (Fig. 1b). The E14-Phy plants had no visible disease symptoms, and the leave morphology was similar to controls plants (Fig. 1a and b). These results indicate that FA is susceptible to PI, MA is tolerant to PI, and E14 is resistant to PI.

### iTRAQ analysis and profile of proteins altered by PI in FA-Phy, MA-Phy, and E14-Phy

**Fig. 2:**
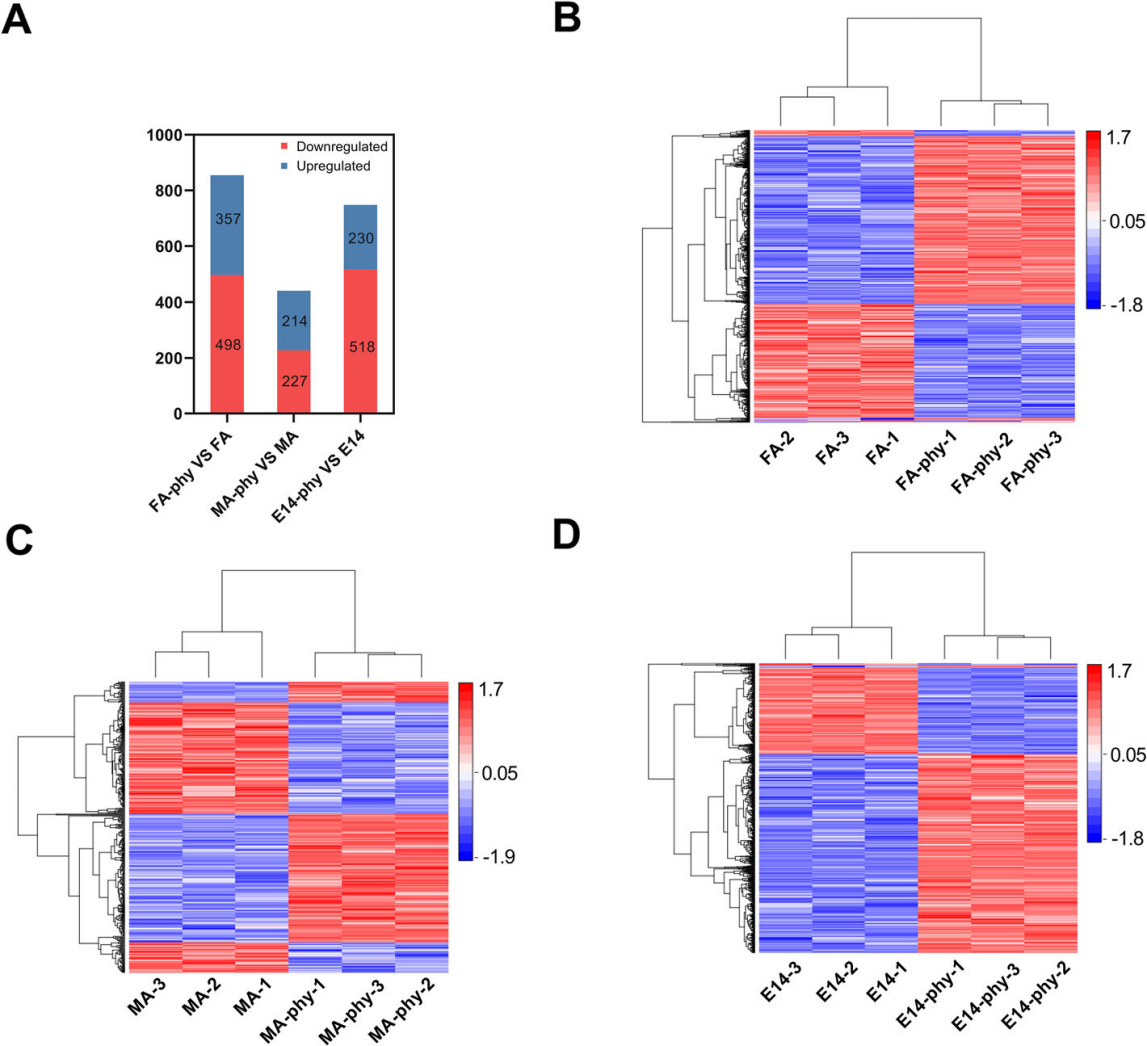
Number of DAPs and their profile between PI treated plants and control. **a** Bar chart showing number of up-regulated and down-regulated proteins in each pairwise comparison of FA-Phy vs. FA, MA-Phy vs. MA and E14-Phy vs. E14. Light blue color indicates down-regulated proteins and navy-blue color indicates up-regulated proteins. **b, c, d** Heat map showing abundance profile of proteins in FA-Phy vs. FA, MA-Phy vs. MA and E14-Phy vs. E14 comparison. Proteins with high abundance (red); proteins with low abundance (blue)

To reveal the molecular response of potato to PI infection at the protein level; we conducted iTRAQ-based proteomics experiments with three potato cultivars FA, MA, and E14 plants infected with PI and controls. We identified a total of 10,689 high-quality, unique peptides corresponding to 3306 proteins, and following the criteria described in the ‘Materials and methods section,’ we quantified 2044 proteins (Additional file 1: Fig. S1, Additional file 2: Table S1, and Additional file 3: Table S2 contains the complete list of identified peptides and proteins, and differentially abundant proteins (DAP) respectively, *p* < 0.05, FC > 1.2, Fig. 2a). Furthermore, Pearson correlation analysis was used to assess the reproducibility of our iTRAQ-based proteomics experiments. The results show a high correlation among the replicates of each sample (Additional file 4: Fig. S2).

The pairwise comparison of the quantified proteins (infected vs. control plants), showed that 855 proteins were differentially abundant in the FA-Phy vs. FA. Out of which 498 proteins were up-regulated, and 357 proteins were down-regulated (Fig. 2b). In MA-Phy vs. MA, 441 proteins showed significant changes in their abundance, of which 227 were up-regulated, and 214 were down-regulated (Fig. 2c). Additionally, the E14-Phy vs. E14 had 748 DAPs, of which 518 were up-regulated, and 230 were down-regulated (Fig. 2d).

**Fig. 3:**
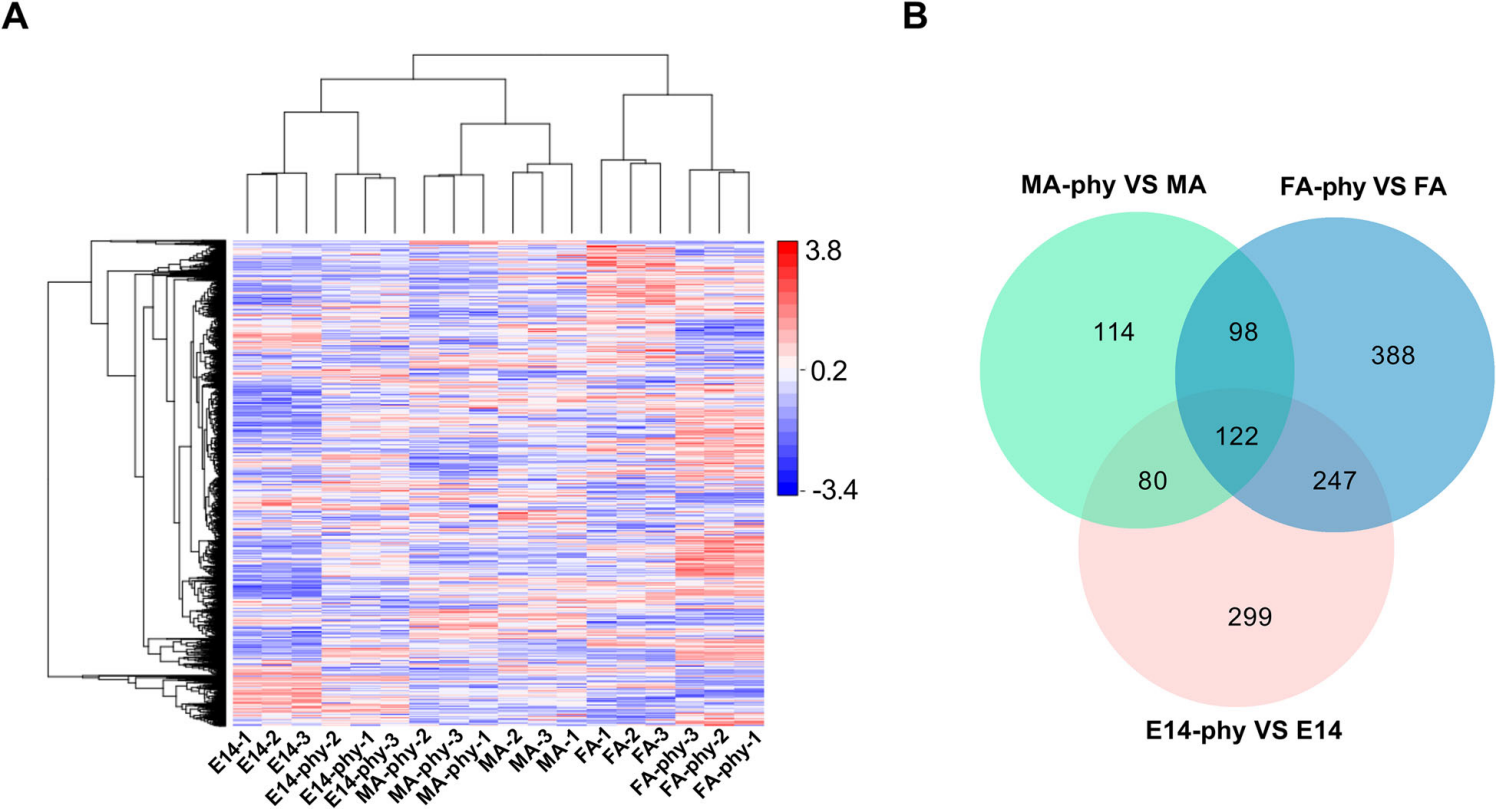
Hierarchical clustering and overlapping proteins. **a** Cluster analysis of differential abundant proteins in FA-Phy vs. FA, MA-Phy vs. MA and E14-Phy vs. E14. Up-regulated proteins (red); down-regulated proteins (blue). **b** Venn diagram representing common and unique differential abundant proteins between FA-Phy vs. FA, MA-Phy vs. MA and E14-Phy vs. E14 comparisons

Cluster analysis of all DAPs showed that proteins of E14-Phy and MA-Phy clustered together separate from FA-Phy (Fig. 3a), suggesting that E14-Phy and MA-Phy have a similar response to PI infection different from FA-Phy. Potato proteins commonly or specifically targeted by PI were identified by overlapping of DAPs in FA-Phy, MA-Phy, and E14-Phy respectively (Fig. 3b). For example, 122 DAPs were shared by the three cultivars, of these, 83 proteins were simultaneously up-regulated, and 24 proteins were consistently down-regulated. Whereas 15 DAPs were dynamically regulated (either up-or-down-regulated in the three cultivars at the same time) in FA-Phy, MA-Phy, and E14-Phy respectively, (Additional file 5: Table S3). In addition to 247 DAPs shared between FA-Phy and E14-Phy, 98 DAPs common to FA-Phy and MA-Phy, and 80 DAPs are shared between MA-Phy and E14-Phy. In contrast, we identified, 338 DAPs exclusively abundant in FA-Phy, of these 235 proteins were up-regulated and 153 DAPs were down-regulated. The MA-Phy, had 141 uniquely abundant DAPs, out of which 58 proteins were up-regulated and 83 proteins were down-regulated, and 299 DAPs were exclusively abundant in the E14-Phy, of which 201 proteins were up-regulated, and 98 were downregulated. Together these results highlight similarities and differences in regulation of protein abundance among the potato cultivars when challenged with PI.

### Gene ontology, enrichment, and pathway analyses

**Fig. 4:**
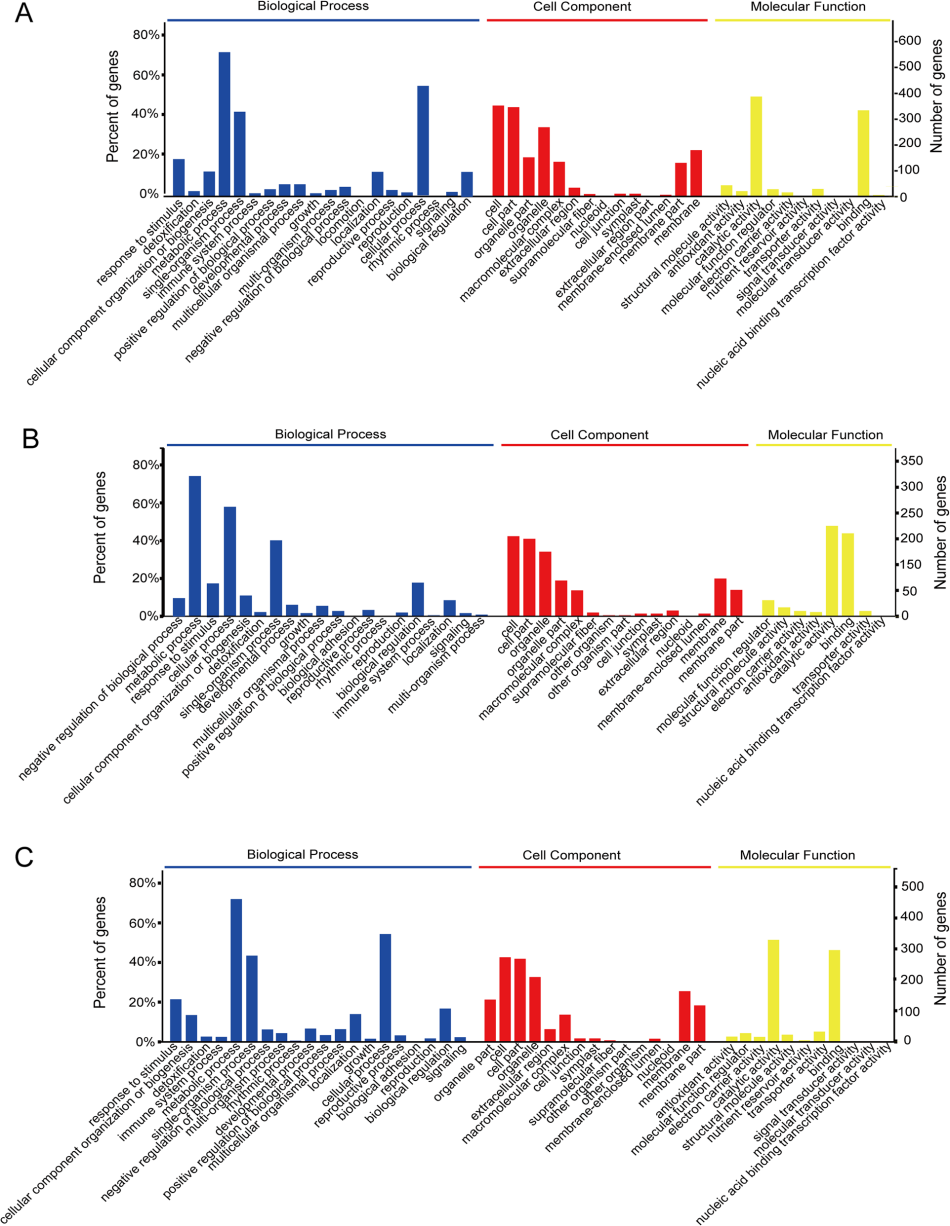
Gene ontology classification of differential abundant proteins identified in FA-Phy vs. FA, MA-Phy vs. MA, and E14-Phy vs. E14 comparison. The results are summarized in terms of three functional: cellular component, molecular function, and biological process. The blue bar represents biological process categories, the red bar represents GO terms for cellular component, and the yellow bar represents biological process categories to molecular function categories

GO terms classification (“biological process,” “molecular function” and “cellular component,” categories) was used to gain information on the biological meaning of differentially abundant proteins (Fig. 4). The results showed that FA-Phy, MA-Phy, and E14-Phy have a similar distribution of GO terms. For example, in FA-Phy, 21 biological processes, 11 molecular functions, and 14 cellular components were altered by PI (Fig. 4a). MA-Phy had 20 biological processes, 15 cellular components, and 8 molecular functions categories (Fig. 4b), and E14-Phy had 20 biological processes, 15 for cellular component, and 11 for molecular functions (Fig. 4). Generally, about 70% of DAPs in the biological process was related to “metabolic process,” and more than 54% of the DAPs related to “cellular process” in each of the cultivar. Similarly, “catalytic,” and “binding” activity was the predominant molecular function shared by the three cultivars. And “cell,” “organelles,” “cellular membrane,” and “macromolecular complex”, were the dominant cellular component among the cultivars.

GO enrichment tests revealed over-represented biological process categories in FA-Phy, MA-Phy, and E14-Phy respectively (Additional file 6: Fig. S3). For example, we found in FA-Phy, significant enrichment of positively regulated biological process categories that include “detoxification,” “stimulus-response,” “metabolic process,” “single-organism process,” and “cellular component organization or biogenesis.” (Additional file 6: Fig. S3a). In contrast, the “immune system process” and “negative regulation of biological process” were negatively enriched (Additional file 6: Fig. S3b).

Similar to FA-Phy, we found “detoxification” and “stimulus-response” to be positively enriched in MA-Phy (Additional file 6: Fig. S3c-e). Nevertheless, we also noticed specific enrichment of “negative regulation of the biological process,” “negative regulation of macromolecule metabolic process,” and “negative regulation of the cellular metabolic process” in MA-Phy. And proteins that fell within these categories have functions associated with “peptidase/endopeptidase inhibitors, and endopeptidase enzyme regulators” (Additional file 6: Fig. S3c). Opposite to negative enrichment of “cellular process” and “developmental process” (Additional file 6: Fig. S3d). Whereas in the E14-Phy, “defense response,” “detoxification,” immune response, and “negative regulation of the biological process” categories showed significant enrichment (Additional file 6: Fig. S3e). In contrast to negative enrichment of “cellular process” and “developmental process.

**Fig. 5:**
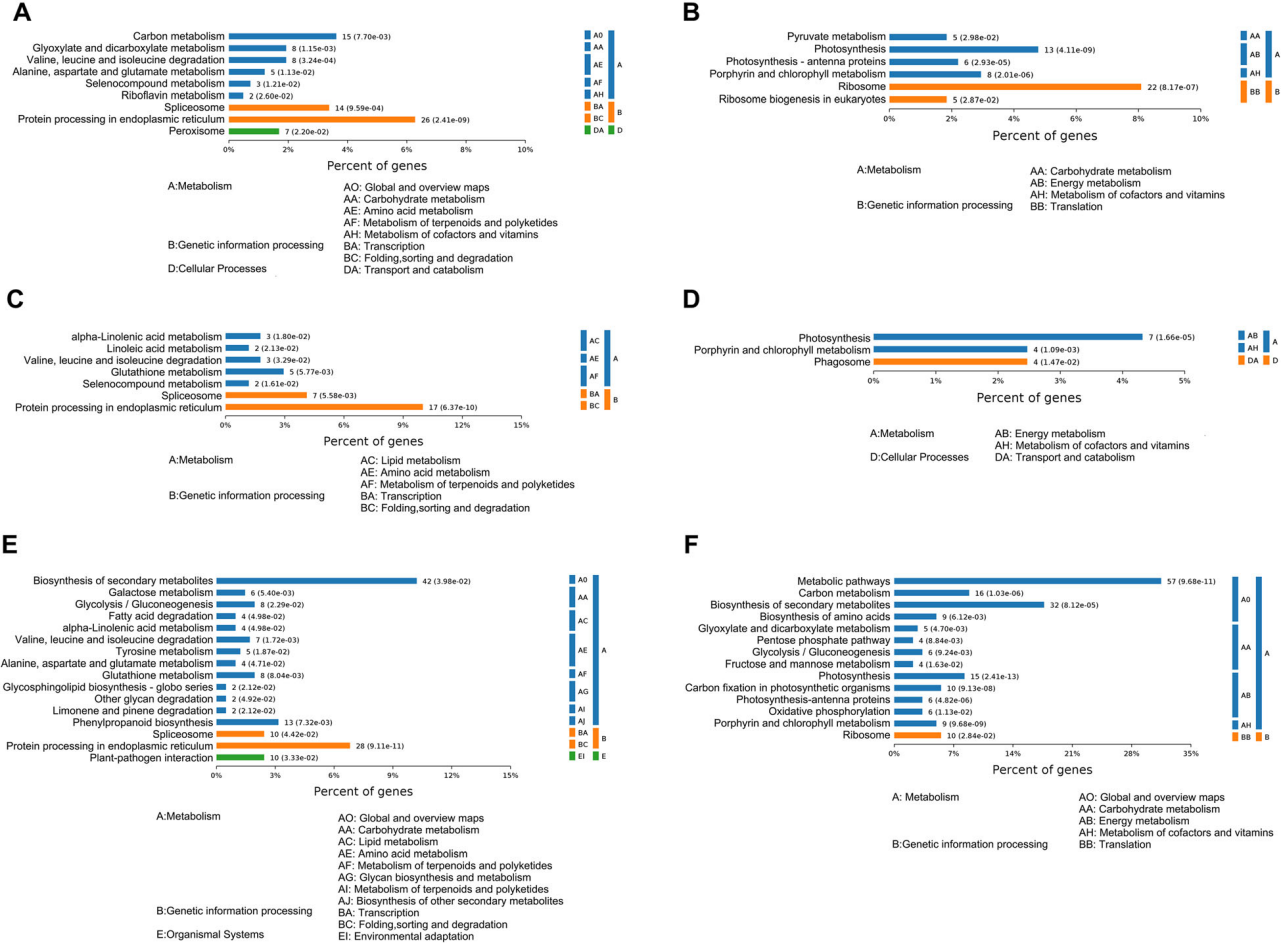
KEGG pathway classification and enrichment tests. **a, b** KEGG enrichment of up-regulated and down-regulated proteins in FA-Phy vs. FA. **c, d** KEGG enrichment of up-regulated and down-regulated proteins in MA-Phy vs. MA. **e, f** KEGG enrichment of up-regulated and down-regulated proteins in E14-Phy vs. E14. The blue bar represents metabolism, orange bar specifies genetic information processing, and green bar represents cellular processes. A/B/C/D/E/F respectively represent main KEGG categories, and A0 AA AE BA BC respectively correspond to the detailed sub-categories in the specific KEGG database

Among the cultivars, the KEGG pathway enrichment analysis further revealed common or specific pathways altered by PI (Fig. 5). For example, “valine, leucine and isoleucine degradation,” “spliceosome,” and “protein processing in the endoplasmic reticulum” were commonly enriched and positively induced in the three cultivars after PI infection.” While “photosynthesis” and “porphyrin and chlorophyll metabolism” are consistently repressed (Additional file 7: Table S4). Alpha-Linolenic and linoleic” and “glutathione metabolism” were specifically enriched and positively induced in MA-Phy and E14-Phy but not FA-Phy. Previous reports suggest that α-linolenic acid or linoleic acid are substrates for LOX and converted into hydroperoxy polyunsaturated fatty acids, which are substrates for many pathways involved in developmental processes and defense including jasmonic acid and salicylic acid, both of which are associated with HR-induced PCD [25]. Also, glutathione metabolism has been linked to the detoxification process and protection against oxidative stress [26]. Additionally, “plant-pathogen interaction pathway,” “phenylpropanoid biosynthesis,” and “biosynthesis of secondary metabolite” pathways were uniquely enriched in E14-Phy infected plants but not MA-Phy and FA-Phy respectively.

The KEGG results suggest that up-regulation of LOX, glutathione metabolism, and plant-pathogen interaction, and phenylpropanoid biosynthetic pathways specific to MA-Phy and E14-Phy respectively might contribute to their phenotype after PI infection.

### Protein-protein interaction in the FA-Phy and E14-Phy

**Fig. 6:**
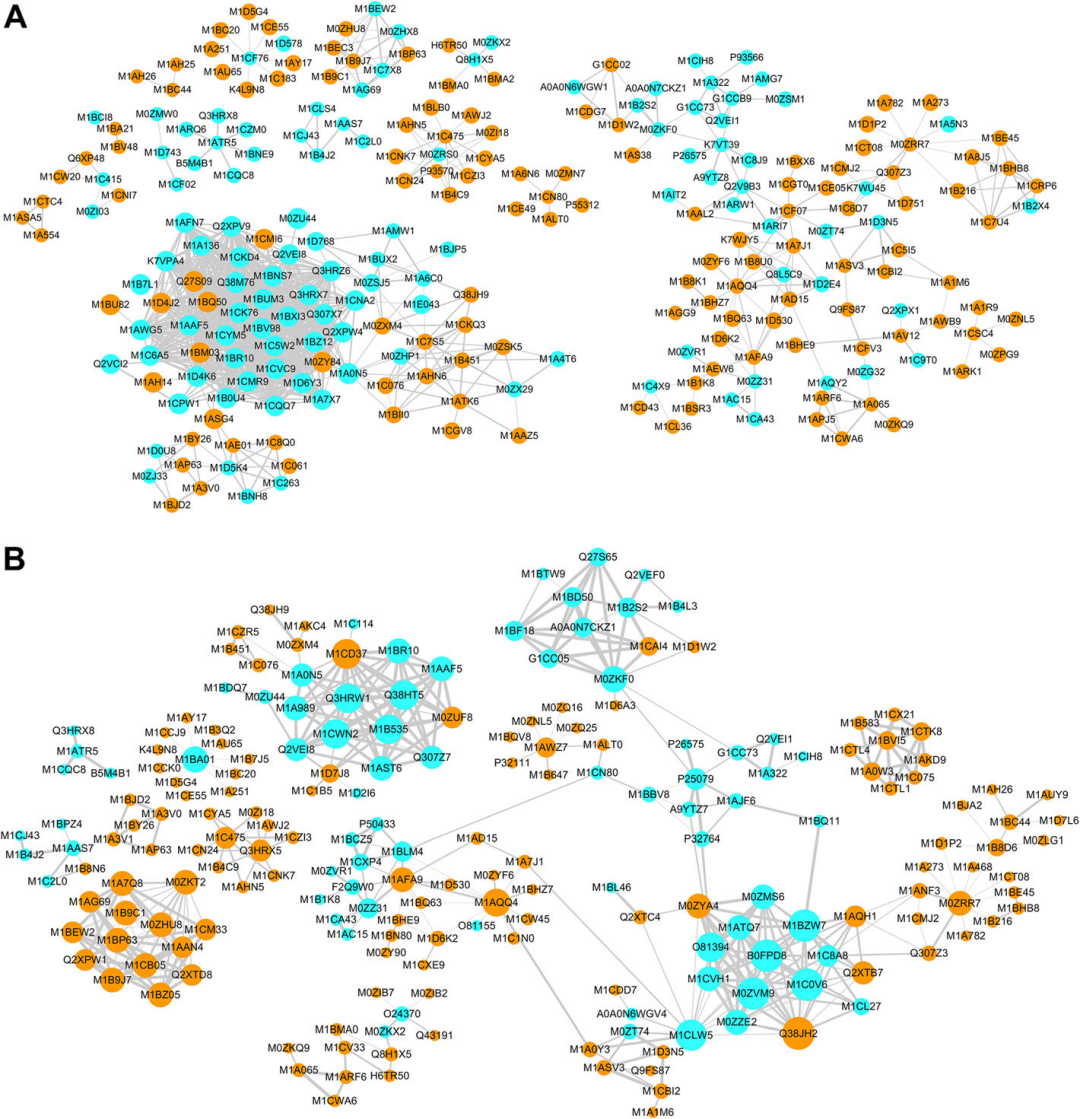
Protein-protein interaction network. **a** Network interactions of differentially regulated proteins in FA after PI infection. **b** Network interactions of differentially regulated proteins in E14 after PI inoculation. Up-regulated proteins are represented by turquoise color and orange color represented down-regulated proteins

To uncover the various functional aspects of potato PI interaction, we analyzed the protein-protein interaction (PPI) that occurred in FA-PI and E14-PI using STRING (http://string-db.org). The PPI network for FA-PI (Fig. 6a) revealed a strong interaction between different protein classes, i.e., photosynthesis, electron transport, translation, ribosome biogenesis, and RNA metabolic process which showed maximum interactions. In opposite, we found very strong interaction among proteins that are involved in defense response, stimulus, protein folding, cellular amino acid metabolic process, biosynthesis of aromatic compounds, and cellular transport in the E14-PI PPI-network (Fig. 6b).

### Validation of differentially abundant proteins by western-blot and qPCR

**Fig. 7:**
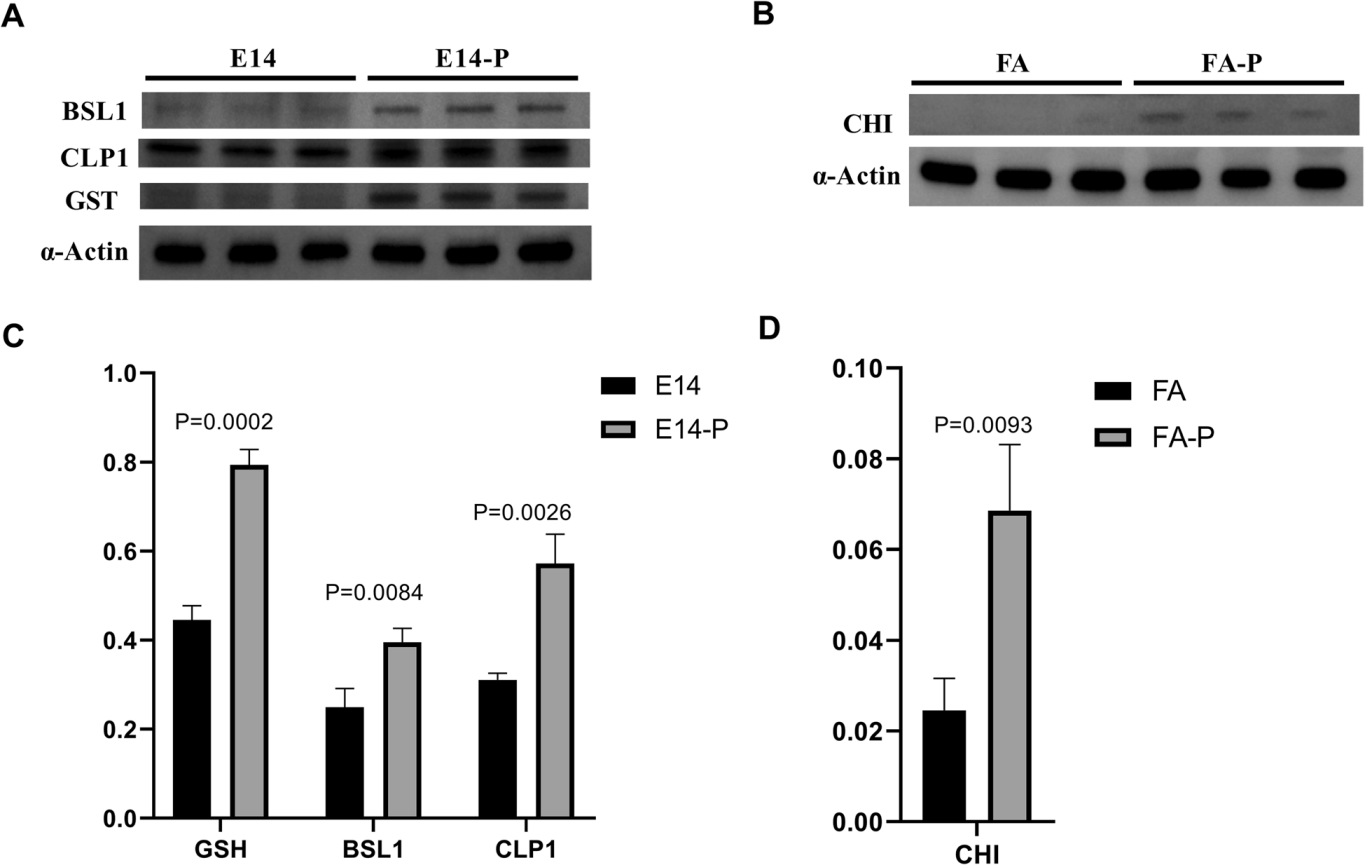
Western blot analysis. Western blot results confirmed protein abundance profile in FA-Phy vs. FA and E14-Phy vs. E14. **a** Western blot analysis showed changes of BSL1, CLP1 and GTS in E14-Phy vs. E14. **b** Western blot analysis showed changes in CHI in FA-Phy vs. FA. **c** Relative foldchange of BSL1, CLP1 and GTS abundance in E14-Phy vs. E14. **d** Relative foldchange of CHI abundance in FA-Phy vs. FA. Potato actin represented loading control

To complement and validate the iTRAQ-based proteomics analysis at the translation level, western blotting assays were performed to check BLS1(Serine/threonine-protein phosphatase), CLP1(Protein CLP1 homolog), GST (Probable glutathione S-transferase, Annexin) level in E14-Phy and CHI (endochitinase) level in FA-Phy (Fig. 7a-d). As shown in Fig. 7a and b, the significant increase in abundance levels of three proteins I6XKY2/BLS1(FC = 1.2), M1CYZ7/CLP1 (FC = 1.22) and P32111/GST (FC = 1.29) and Q2HPK8/CHI (FC = 1.38) from ITRAQ analysis was consistent with western blot results for example P32111/GST increase about two-folds (from 0.45 to 0.8, *p* = 0.0002) in E14-Phy compared to E14, also I6XKY2/BLS1 was upregulated in infected plants (from 0.25 to 0.4, *p* = 0.0084), and M1CYZ7/CLP1 increased more than 1.5 folds in abundance (from 0.3 to 0.6p = 0.0026) (Fig. 7c). Similarly, the abundance of Q2HPK8/CHI (FC = 1.38) (Fig. 7b) was two-folds higher in FA-Phy compared to FA from 0.25 to 0.65, *p* = 0.0093) (Fig. 7d). Potato actin represented loading control use to normalized band intensity for the proteins, the original gel images are reported in Additional file 8, Supplementary Fig. S4.

**Fig. 8:**
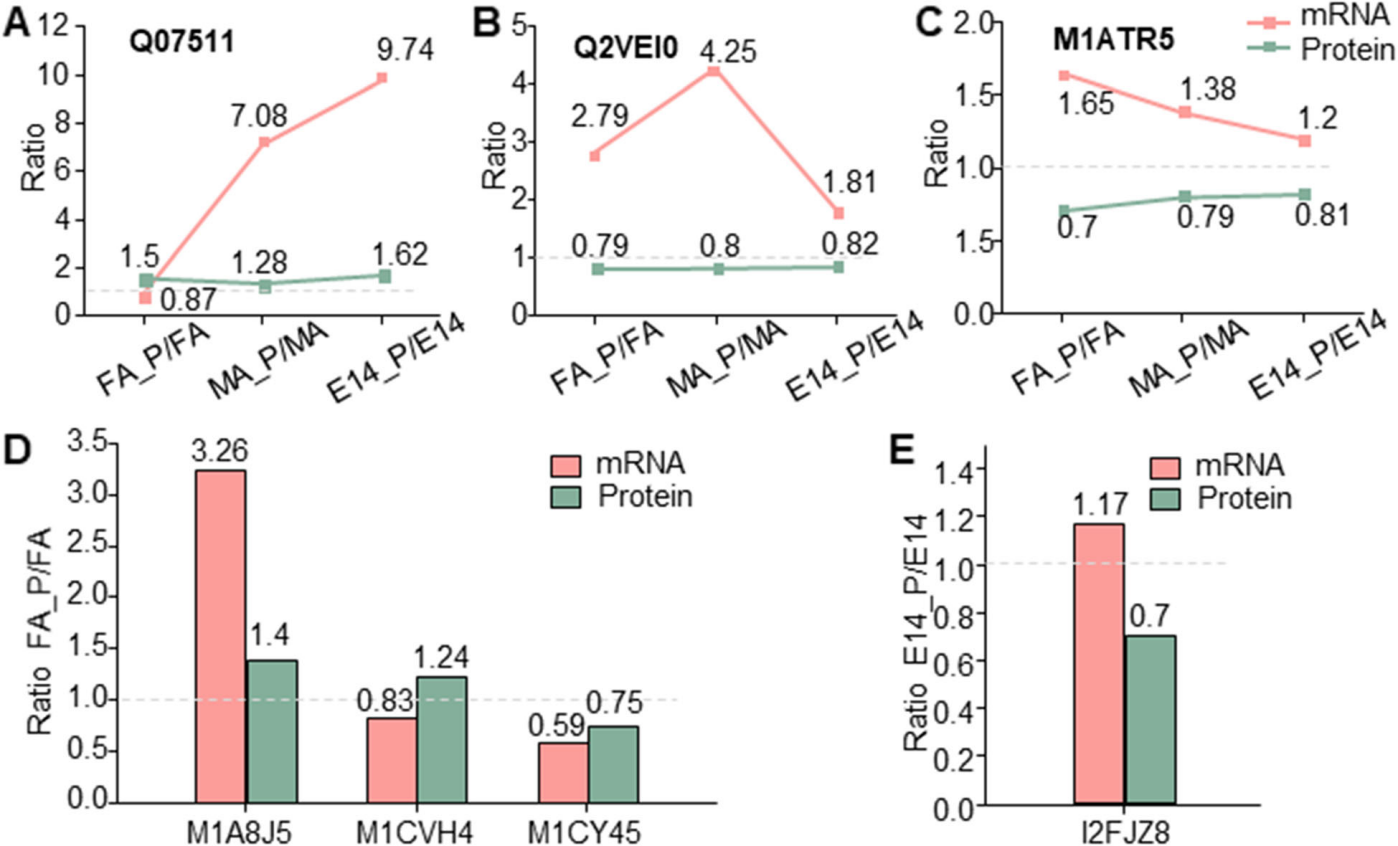
Real-time quantitative PCR analysis. qPCR results of selected up- and down-regulated genes. **a** mRNA expression levels of three proteins randomly selected from iTRAQ data set. **b** mRNA expression levels of three proteins randomly selected from FA-Phy vs. FA. **c** mRNA expression levels of one protein randomly selected from E14-Phy vs. E14. The green bar and line indicate the protein abundance determined by iTRAQ and orange bar shows relate expression of mRNA. All data are presented as mean ± SD (*n* = 3)

The qPCR analysis was used to confirm the ITRAQ data at the transcript level. We analyzed the relative expression pattern of genes encoding seven representative DAPs. The selected proteins were involved in multiple biological processes, including the stress and defense process, cellular metabolic process, signaling, and transport. As shown in Fig. 8, positive trend correlations between protein and mRNA expression levels were detected for Q07511, M1A8J5, and M1CY45 which suggest that the abundance of these proteins is likely regulated at the transcriptional level. However, the abundance of the Q2VEI0, M1ATR5, M1CVH4, and I2FJZ8 transcripts was the opposite of their protein abundance suggests further regulation of these transcripts probably due to posttranslational modifications.

## Discussion

### The profile of shared proteins revealed a common response to PI among the potato cultivars

Locally induced plant responses to pathogenic fungus include accumulation of reactive oxygen species (ROS), hypersensitive reaction (HR), and production of pathogenesis-related (PR) proteins [5, 27]. Our iTRAQ analysis identified proteins shared among the cultivars that have functions related to ROS, HR, and PR respectively. They include two peroxidases (M1AY17 and M1BC20), Endochitinase (Q6B782), a probable linoleate 9S-lipoxygenase 5 (Q43191), LRR receptor-like kinase (A7UE73), small heat shock protein (K7VKA6), and pathogenesis 2-related protein (K7VK61/PR), and four glycosidases (M0ZHI6, M1D7B3, K9MBH7, and P52401), (Table 1). Analysis of protein abundance indicates that these proteins on the average were 1.5-folds higher in abundance compared to control and were consistently up-regulated in the three cultivars (Table 1). Furthermore, GO analysis revealed that these common up-regulated proteins played a role in stress response and defense-related processes.

**Table 1:** Proposed candidate protein functionally related to susceptible, tolerance, and resistance of late blight disease in potato

Accession	Description	FA-Phy		MA-Phy		E14-Phy	
		Fold Change	***P*** value	Fold Change	*P* value	Fold Change	*P* value
**Common DAPs up-regulated in FA-Phy, MA-Phy and E14-Phy**							
K7VKA6	17.6 kDa class 1 small heat shock protein	3.82	0.00	3.67	0.00	2.54	0.00
K7VK61	Pathogenesis-related protein P2	1.35	0.00	1.46	0.00	2.53	0.00
M1AY17	Peroxidase	2.37	0.00	1.29	0.00	2.34	0.00
M1BC20	Peroxidase	1.36	0.00	1.27	0.00	1.41	0.00
Q6B782	Endonuclease	2.50	0.00	1.70	0.02	2.77	0.00
Q43191	Probable linoleate 9S-lipoxygenase 5	2.02	0.00	1.47	0.00	2.25	0.00
A7UE73	LRR receptor-like kinase	1.44	0.00	1.21	0.04	1.70	0.00
M0ZHI6	Beta-galactosidase	1.91	0.00	1.35	0.00	1.75	0.00
M1D7B3	Beta-galactosidase	2.38	0.00	1.23	0.03	2.21	0.00
K9MBH7	Beta-1,3-glucanase 22	2.30	0.00	1.27	0.00	2.10	0.00
P52401	Glucan endo-1,3-beta-glucosidase, basic isoform 2	2.46	0.00	1.39	0.02	2.79	0.00
**Common DAPs down-regulated in FA-Phy, MA-Phy and E14-Phy**							
G1CC73	Photosystem II protein D1	0.82	0.00	0.74	0.00	0.83	0.00
Q2VEI0	Photosystem II CP43 reaction center protein	0.79	0.00	0.80	0.00	0.82	0.00
M1AY18	Chlorophyll a-b binding protein, chloroplastic	0.76	0.00	0.81	0.00	0.65	0.00
M1A322	Cytochrome b559 subunit alpha	0.83	0.00	0.81	0.01	0.70	0.00
Q93XJ9	Ferredoxin	0.80	0.02	0.76	0.00	0.63	0.00
**Protein reprogrammed in FA-Phy, MA-Phy and E14-Phy**							
M1BBH5	Non-specific lipid-transfer protein	1.39	0.01	0.72	0.01	1.25	0.02
M1BNE8	Uncharacterized protein	1.34	0.00	0.77	0.00	0.75	0.00
P01052	Chymotrypsin inhibitor I, A, B and C subunits	0.62	0.00	1.70	0.00	0.57	0.00
Q8H0L9	DS2 protein	0.58	0.01	1.22	0.00	0.62	0.00
Q43834	Class II chitinase	0.78	0.03	1.60	0.00	1.77	0.00
M1LA62	Kunitz-type protease inhibitor D (Fragment)	0.74	0.00	1.95	0.00	1.21	0.00
M1AG69	Histone H2B	0.78	0.00	1.30	0.00	1.27	0.00
Q8H1X5	Allene oxide cyclase	0.83	0.00	0.79	0.00	1.20	0.00
M1D768	Uncharacterized protein	0.64	0.00	0.68	0.00	1.34	0.00
M1D478	Uncharacterized protein	0.65	0.00	0.53	0.00	1.32	0.00
**FA specific proteins altered by PI**							
M1CLH3	Uncharacterized protein containing PR-like domain	1.31	0.03	–	–	–	–
M1BN73	Uncharacterized protein containing PR-like domain	0.75	0.00	–	–	–	–
M1DR90	Uncharacterized protein containing PR-like domain	0.52	0.00	–	–	–	–
M1AJH8	Uncharacterized protein containing PR-like domain	0.55	0.05	–	–	–	–
M0ZJT7	Uncharacterized protein containing PR-like domain	0.80	0.00	–	–	–	–
M1BAU5	Uncharacterized protein containing PR-like domain	0.63	0.02	–	–	–	–
M1C4P4	Uncharacterized protein containing PR-like domain	0.60	0.00	–	–	–	–
M1BAU6	Uncharacterized protein containing PR-like domain	0.75	0.00	–	–	–	–
M1ACY3	Uncharacterized protein containing PR-like domain	0.58	0.01	–	–	–	–
Q307X7	Ribosomal protein PETRP-like	0.81	0.00	–	–	–	–
Q2XPV9	40S ribosomal protein S8	0.78	0.00	–	–	–	–
Q3HRZ6	40S ribosomal protein S8	0.78	0.00	–	–	–	–
Q3HRX7	Ribosomal protein L25-like protein	0.77	0.01	–	–	–	–
M1D4K6	Ribosomal protein L19	0.73	0.00	–	–	–	–
K7VPA4	Ribosomal protein L24	0.70	0.00	–	–	–	–
Q2XPW4	60S ribosomal protein L7A-like protein	0.69	0.00	–	–	–	–
M1CGC9	3-phosphoshikimate 1-carboxyvinyltransferase	0.78	0.00	–	–	–	–
M1BTT7	Ketol-acid reductoisomerase	0.78	0.00	–	–	–	–
M1AIT2	D-3-phosphoglycerate dehydrogenase	0.79	0.00	–	–	–	–
M1CZC0	ERBB-3 BINDING PR	0.81	0.00	–	–	–	–
A0A0M4KNM3	Carotenoid 9,10(9′,10′)-cleavage dioxygenase 1-like protein (Fragment)	0.77	0.00	–	–	–	–
Q9ZRB6	Ci21A protein	0.38	0.00	–	–	–	–
K7VKB1	TAS14 peptide	0.42	0.00	–	–	–	–
**MA specific proteins induced against PI**							
A0A097H183	PIN-I protein	–	–	2.24	0.00	–	–
Q3S492	Proteinase inhibitor I	–	–	1.47	0.00	–	–
A0A097H193	PIN-II protein	–	–	1.44	0.03	–	–
E0WCF2	Type I serine protease inhibitor	–	–	1.85	0.00	–	–
J7EQ46	Proteinase inhibitor II type C-b	–	–	1.73	0.00	–	–
Q41434	Wound-inducible proteinase inhibitor I (Fragment)	–	–	1.38	0.00	–	–
M1BSA4	Carboxypeptidase	–	–	1.35	0.00	–	–
P37842	Multicystatin	–	–	1.94	0.00	–	–
A0A097H108	KTI-A protein (Fragment)	–	–	1.47	0.01	–	–
A0A097H114	KTI-A protein	–	–	1.34	0.00	–	–
M0ZWN2	Thioredoxin	–	–	1.23	0.00	–	–
Q3HVN5	Dehydroascorbate reductase	–	–	1.30	0.04	–	–
A2ICR9	Dehydroascorbate reductase	–	–	1.23	0.00	–	–
A9LMM9	Dehydroascorbate reductase	–	–	1.22	0.00	–	–
P31212	Threonine dehydratase biosynthetic (Fragment)	–	–	1.31	0.00	–	–
M1BC24	Phospho-2-dehydro-3-deoxyheptonate aldolase	–	–	1.24	0.02	–	–
G9IHI3	Apoplastic invertase	–	–	0.80	0.00	–	–
F2Q9V9	Glyceraldehyde-3-phosphate dehydrogenase	–	–	0.82	0.01	–	–
M1ALJ6	Phosphotransferase	–	–	0.83	0.03	–	–
M1BQC2	Pectinesterase	–	–	0.82	0.00	–	–
Q38JH8	S-adenosylmethionine synthase 2	–	–	0.73	0.00	–	–
M1CD27	Methylthioribose-1-phosphate isomerase	–	–	0.79	0.01	–	–
M1BTK3	Potassium transporter	–	–	0.74	0.01	–	–
M1BM79	Ammonium transporter	–	–	0.76	0.00	–	–
**E14 specific proteins deployed against PI**							
P52403	Endochitinase 1 (Fragment)	–	–	–	–	3.56	0.00
O81144	Class I chitinase	–	–	–	–	1.33	0.01
Q84XG7	Erwinia induced protein 1	–	–	–	–	1.26	0.00
M0ZG93	Mitogen-activated protein kinase	–	–	–	–	1.20	0.01
M1B7J5	Peroxidase	–	–	–	–	1.41	0.00
M1B3Q2	Peroxidase	–	–	–	–	1.39	0.00
M1CCK0	Peroxidase	–	–	–	–	1.31	0.00
M1CCJ9	Peroxidase	–	–	–	–	1.23	0.00
Q38JB4	Chloroplast lipocalin	–	–	–	–	1.22	0.01
P32111	Probable glutathione S-transferase	–	–	–	–	1.29	0.00
Q84U63	Osmotin-like protein (Fragment)	–	–	–	–	1.87	0.04
Q5XUG9	Putative thaumatin-like protein	–	–	–	–	1.43	0.01
Q8LRU6	Pathogenesis related protein 10 (Fragment)	–	–	–	–	1.38	0.03
A0A097H100	Clone PI9650 defensin-like protein mRNA	–	–	–	–	1.46	0.00
M1BC19	RSI1	–	–	–	–	1.21	0.02
M1BV78	Peptidylprolyl isomerase	–	–	–	–	1.22	0.00
Q2XTE5	Hsp90–2-like	–	–	–	–	1.31	0.04
Q3HRX5	DnaJ-like protein	–	–	–	–	1.32	0.02
I6XKY2	Serine/threonine-protein phosphatase	–	–	–	–	1.24	0.01
M1CXE9	Uncharacterized protein	–	–	–	–	1.44	0.00
M1C047	Uncharacterized protein	–	–	–	–	1.47	0.01
M1AZW1	Calcium-transporting ATPase	–	–	–	–	1.22	0.00
M1BXT8	Calcium-transporting ATPase	–	–	–	–	1.36	0.00
M0ZSI1	Importin subunit alpha	–	–	–	–	1.37	0.00
M1B7C9	Importin subunit alpha	–	–	–	–	1.22	0.02
M1C203	Vacuolar protein sorting-associated protein 35	–	–	–	–	1.42	0.00
M1C203	Vacuolar protein sorting-associated protein 35	–	–	–	–	1.42	0.00
M1DLL0	Protein transport protein Sec61 subunit beta	–	–	–	–	1.20	0.00

Among the shared up-regulated proteins, of interest were the peroxidases, endochitinase, PR protein, LRR receptor-like kinase protein, because of their abundance in the three cultivars. For example, Endochitinase usually acts as part of fungal elicitor and plant defense signaling component [24], peroxidases are implicated in pathogen-induced oxidative stress and activation of defense-related activities in potato [28], whereas the LRR receptor-like kinase proteins are involved in perception, recognition, and transmission of external stimulus through signaling cascades to elicit appropriate cellular responses to pathogenic invasion [29]. In the present study, the abundance of A7UE73 was higher in the E14-Phy compared to its lower level FA-Phy (Table 1), suggesting weak pathogen recognition in FA-Phy.

Among the shared proteins, we found that proteins related to photosynthesis and “electron transport chain were specifically down-regulated (Table 1). They include three photosystem II proteins (G1CC73, Q2VEI0, M1AY18), chlorophyll a-b binding protein, chloroplastic (M1A322), and ferredoxin (Q93XJ9). The suppression of these proteins is consistent with previous studies which show that proteins related to photosynthetic pathways are down-regulated in potato during PI invasion [24, 30].

### Dynamic reprogramming of shared proteins revealed potential cultivar specific reaction to PI

Overlap of the iTRAQ data set revealed15 proteins shared by the cultivars which were reprogrammed after PI infection (Table 1). Among them, a non-specific lipid transfer protein (M1BBH5) and an uncharacterized protein belonging to the ataxin-3 family (M1BNE8), were up-regulated in FA-Phy but down-regulated in MA-Phy. The class II chitinase (Q43834), chymotrypsin inhibitor (P01052), Kunitz-type protease inhibitor KTI (M1LA62), abscisic stress/wound-induced protein DS2 (Q8H0L9), and Histone H2B (M1AG69), were up-regulated in MA-Phy but repressed in FA-Phy. At the same time, chymotrypsin inhibitor, and DS2 protein were down-regulated in E14-Phy. Here, our result showed that the abundance of protease inhibitors and wound-induced proteins in MA-Phy correlates with host response against pathogenic infection [31, 32]. Furthermore, allene oxide cyclase (Q8H1X5) and two uncharacterized proteins M1D768 and M1D478 containing alpha/beta knot methyltransferases domain were consistently down-regulated in FA-Phy and MA-Phy, opposite to their high induction in the E14-Phy. Previous studies have shown that allene oxide synthase (AOS) catalyzes the first reaction leading to the formation of jasmonates and jasmonic acid (JA). JAs are known to mediate defense responses against pathogenic fungus [33, 34]. Here, it is likely that JA canonical pathway was highly induced E14-Phy and might contribute to the resistant phenotype of E14-Phy in contrast to FA-Phy. Together, these results highlight the difference in each cultivar response to PI.

### FA proteins specifically targeted by late blight disease pathogen *P. infestans*

Analysis of FA proteome response to PI infection revealed specific repression of defense proteins, proteins involved in primary metabolism, and hormone signaling process. For instance, nine uncharacterized proteins (M1CLH3, M1BN73, M1DR90, M1AJH8, M0ZJT7, M1BAU5, M1C4P4, M1BAU6, and M1ACY3) containing pathogenesis-related protein Bet v I (PR) binding domain were specifically down-regulated in FA-Phy with very high negative foldchange (Table 1). PR-related proteins are antimicrobial proteins induced by the host against the pathogen [35]. The suppression of PR proteins suggests that PI compromised the FA-Phy defense system.

Additionally, proteins that constitute ribosomal subunits such as PETRP-like (Q307X7), 40S-RPS8 (Q2XPV9, and Q3HRZ6), RPL25-like protein (Q3HRX7), RPL19 (M1D4K6), RPL24 (K7VPA4), and 60S RPL7A-like protein (Q2XPW4), were suppressed by PI in FA-Phy (Table 1). One study suggests that RPL12 and RP-L19 play a role in non-host resistance against the pathogen [36]. In the present study, the downregulation of many ribosomal proteins suggests that PI diminished basal defense mechanism in FA-Phy, which is in agreement with the phenotype of FA-Phy after infection.

Also, we noticed specific suppression of proteins related to aromatic amino acid biosynthesis and metabolisms such as (phosphoshikimate 1-carboxyvinyltransferase (M1CGC9), ketol-acid reductoisomerase (M1BTT7), and D-3-phosphoglycerate dehydrogenase (M1AIT2). The phosphoshikimate 1-carboxyvinyltransferase is a key enzyme of the shikimate pathway, involved in the biosynthesis of multiple aromatic compounds, including chorismite, phenylalanine, tyrosine, and tryptophan [37]. The ketol-acid reductoisomerase belonged to the family of nicotinamide adenine dinucleotide phosphate (NADPH)-dependent oxidoreductases, involved in the supply of flux for the metabolism of valine, isoleucine, and leucine [38]. Whereas D-3-phosphoglycerate dehydrogenase catalyzes the reversible oxidation of 3-phospho-D-glycerate to 3-phosphonooxypyruvate, the committed step in the pathway of L-serine biosynthesis [39].

Typically, hormones play a vital role in defense/immunity against pathogenic invasion, however, various pathogens also manipulate hormone signaling pathways to alter the plant defense response. In FA-Phy, hormone signaling pathways were also not left out in the onslaught by PI. For instance, we observed a specific down-regulation of ERBB-3 binding protein 1 (M1CZC0/EBP-1) and carotenoid (9′,10′)-cleavage dioxygenase 1-like enzyme, (A0A0M4KNM3) involved in hormone signaling. The EBP-1 regulates auxin-mediated signal transduction, which promotes growth and development [40], and 9-cis-epoxycarotenoid dioxygenase is an important enzyme for abscisic acid (ABA) biosynthesis [41]. Similarly, two peptides Ci21A and TASI14 (Q9ZRB6 and K7VKB1) involved in ABA-mediated water stress and desiccation [42] were also down-regulated by PI.

A growing body of evidence indicates that R genes products require the combined effect of, pathogenesis-related proteins, hormones, and phenolic compounds in a dose-dependent manner to provide superior or durable resistance against PI [43]. The present results suggest that the significant reduction of PR proteins, as well as the repression of precursors for biosynthesis of hormones and phenolic compounds, promoted PI pathogenesis in FA-Phy, which is in agreement with FA-Phy phenotype (Fig. 1).

### MA specific response to late blight disease pathogen *P. infestans*

A typical outcome of potato-PI incompatible interaction is the development of localized HR [24, 27]. In this study, MA-Phy developed few macroscopic HR lesions compared to FA-Phy (Fig. 1), probably due to the induction of antifungal proteins uniquely specific to MA. Indeed, most of the defense-related proteins were positively induced and include a group of protease inhibitors and antifungal peptides such as PIN-I (A0A097H183 and Q3S492), PIN-A (A0A097H193), PIN-II (E0WCF2), PIN II- type C-b (J7EQ46), wound-inducible proteinase inhibitor I (Q41434), carboxypeptidase enzyme (M1BSA4), multicystatin (P37842), and Kunitz-type soybean trypsin inhibitor (KTI) protein (A0A097H108 and A0A097H114) (Table 1). The accumulation of several protease inhibitors in MA-Phy is consistent with reports that suggest a battery of proteolytic enzyme inhibitors, enzyme regulators, and peptidase inhibitors are up-regulated to overcome PI during potato-PI incompatible interaction [43–45]. Also, enzymes involved in antioxidant activity accumulated in MA-Phy, for example, thioredoxin (M0ZWN2) and dehydroascorbate reductases (Q3HVN5, A2ICR9, and A9LMM9) were highly up-regulated. Thioredoxins participate in the defense against cellular oxidative damage [46], and dehydroascorbate reductases are involved in scavenging radicals and non- oxygen radicals [47].

Several proteins involved in secondary metabolism were positively induced, but we focused on two proteins threonine dehydratase (P31212) and Phospho-2-dihydro-3-deoxyheptonate aldolase (M1BC24) related to the shikimate pathway. Threonine dehydratase is the first enzyme in L-isoleucine biosynthesis, catalyzing deamination and dehydration of threonine to produce 2-ketobutyrate and ammonia. One report indicates that this enzyme is induced in response to wounding, abscisic acid, and jasmonic acid signaling [48]. The Phospho-2-dihydro-3-deoxyheptonate aldolase catalyzes the first step of chorismate biosynthesis and is a part of metabolic intermediates that provides precursors to the phenylpropanoid pathway for the biosynthesis of phenolic compounds.

In contrast, many proteins involved in cellular processes were repressed in MA-Phy. For example, apoplastic invertase (G9IHI3), glyceraldehyde-3-phosphate dehydrogenase (F2Q9V9/GAPDH), phosphotransferase (M1ALJ6), and pectinesterase (M1BQC2). The apoplastic invertase is a member of the glycoside hydrolase family that catalyzes the hydrolysis of sucrose into fructose and glucose, (Niki et al., 1998). The GAPDH is involved in the breakdown of glucose to produce energy and carbon molecules [49]. The suppression of GAPDH could lead to a reduction in the carbon source available for pathogenic growth. The phosphotransferases belong to the hexokinase family and are involved in cellular glucose homeostasis [50]. The pectinesterase is a cell wall modifying enzyme, involved in pectin degradation [51]. The significant decrease in abundance of these carbon and energy-generating enzymes could be part of a MA defense system to contain the invading pathogen.

Interestingly, we found that S-adenosylmethionine synthase 2 (Q38JH8) and methylthioribose-1-phosphate isomerase (M1CD27) were also repressed. Both enzymes are critical components of the methionine salvage system which can be used for the production of ethylene, cysteine, and other sulfur-containing amino acids [52]. Sauter et al., [53] reported that pathogenic microbes are capable of exploiting a range of organic and inorganic sulfur within the host. In the present study, the depression of enzymes of the methionine salvage pathway resembles a host defense mechanism geared towards restricting PI growth by reducing sulfate sources. Furthermore, methionine is a precursor for the biosynthesis of ethylene and other polyamines [54], although the role of ethylene in plant-pathogen interaction is still unclear. One report suggests that pathogens manipulate ethylene biosynthesis to promote pathogenicity in host plants [40]. For example, ethylene was shown to promote compatible interaction between *P. pyrifolia* and a necrotrophic fungus *Alternaria alternata* [55]. But, in other pathosystems, ethylene was shown to mediate incompatible interaction [40]. In this study, therefore, it is likely that PI infection resulted in host attenuation of ethylene biosynthesis in MA.

To be successful, most fungal pathogens exploit sources of the nutrients within their host to support growth. In the MA-Phy, two transport proteins: potassium transporter (M1BTK3) and ammonia transporter (M1BM79) were down-regulated. Ammonium and urea are essential nitrogen sources for many pathogens, including fungus, and the accumulation of these compounds has been associated with pathogenicity [56]. Here, the decrease in abundance of nutrient transporters by the host seems to be part of the MA defense strategy for suppressing PI growth.

Together these results highlight the potential role of protease inhibitors, antioxidants, aromatic compounds in MA defense protein machinery against PI opposite to the phenotypic and molecular response observed in FA.

### E14 specific response to late blight disease pathogen *P. infestans*

Evaluation of E14 proteome specific response to PI infection revealed strong pathogen recognition and efficient activation of immune response against PI. Proteins responsible for PI recognition and defense signaling components were up-regulated in E14-Phy after infection. In particular, the two endochitinases (P52403 and O81144), erwinia induced protein 1 (Q84XG7/Ei1), and mitogen-activated protein kinase (M0ZG93/MAPK) (Table 1). Generally, chitinases are typically induced as a part of defense machinery against chitin-containing fungal pathogens [57]. Ei1 contained the LysM domain, a small globular domain that can bind peptidoglycan and chitin containing microbes [58]. Here, we suspect that Ei1 might be a specific elicitor or PI recognition factor in E14-Phy. The MAPKs are prominent defense signaling proteins involved in the transduction of specific immune reaction against PI [59].

The proteins involved in scavenging of reactive oxygen species (ROS) and detoxification of microbial compounds were also specifically abundant and perhaps up-regulated in response to PI by E14-Phy (Table 1). They include four peroxidases; (M1B7J5, M1B3Q2, M1CCK0, and M1CCJ9), a chloroplast lipocalin protein (Q38JB4), a probable glutathione S-transferase (P32111/GSTs), osmotin-like protein (Q84U63), thaumatin-like protein (Q5XUG9), and pathogenesis-related protein 10 (Q8LRU6/PR-10). GSTs have an antioxidative effect against oxidative stress [60] and the abundance of the protein was confirmed by western blot. The osmotin and thaumatin produce antifungal activity [61], and PR proteins are highly induced during microbial infection and wounding [62].

Like in the MA-Phy, several protease inhibitors accumulated in E14-Phy after PI infection (Table 1). The most prominent among them is the Clone PI9650 defensin-like protein mRNA (A0A097H100), an antifungal peptide that belongs to the defensin family, and involved in innate immune system response directed primarily against fungal pathogens [63]. Another defense-associated protein specifically induced by PI infection is the RSI1 (M1BC19). RSI1 is a membrane-bound protein similar to prohibitin and contains a band7 domain. The specific function of the RSI1- band7 domain during potato-*P. infestans* interaction is unclear. However, in Arabidopsis, RSI1 was shown to encode putative histone demethylase that interacts with GSTT2 and WRKY transcription factors to activate systemic acquired resistance (SAR) against the pathogen [60]. We equally noticed the induction of peptidylprolyl isomerase PPIase (M1BV78). PPIases are categorized as immunophilins/immunosuppressive ligands, that mediate protein-to-protein interaction, as well as heat-stress, and pathogen virulence-associated factors [64]. Here, the direct role of PPlase during E14-PI interaction is unclear, however, we speculate that it might be involved in the protein modification process or participate in the signal transduction of immune response.

Furthermore, proteins associated with heat stress, hormone signaling, and transcription factor activity were also abundant in E14-Phy (Table 1). For example, two heat shock binding proteins (Q2XTE5 and Q3HRX5/HSPs), serine/threonine-protein phosphatase (I6XKY2/ BLS1), and two uncharacterized proteins containing Ornithine aminotransferase (M1CXE9/OAT) and a WRKY (M1C047) domain respectively. HSPs maintain the folding of newly synthesized proteins, stabilization, and refolding of denatured proteins during heat stress [65]. The BLS1 is a member of the phosphoprotein phosphatase (PPP) family, involved in multi-diverse processes including brassinosteroid signaling, auxin signaling, ROS signaling, and defense response [66]. The OAT is involved in proline biosynthesis and proline is implicated in multiple defenses and stresses tolerance process, including hormone signaling and programmed cell death [67]. WRKY transcription factors regulate diverse physiological processes, including pathogen defense, stress responses, senescence, trichome development, and the biosynthesis of secondary metabolites [68].

**Fig. 9:**
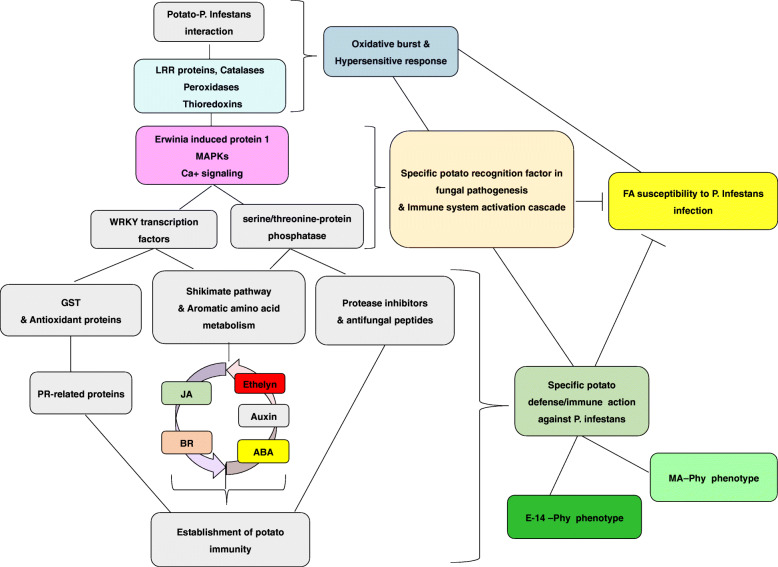
Proposed model of Potato-*P. infestans* interaction. The model showed FA, MA and E14 interaction with PI and highlighted specific repression and accumulation of defense proteins in the three cultivars

Additionally, several proteins related to transport were equally abundant in E14-Phy. They include two calcium-transporting ATPase (M1AZW1 and M1BXT8), two Importin subunit alphas (M0ZSI1and M1B7C9), vacuolar protein sorting-associated protein (M1C203/VSP), and transport protein Sec61 subunit beta (M1DLL0) (Table 1). GO analysis showed that the calcium-transporting ATPases are involved in ATP coupled calcium transmembrane transport. Many studies have linked calcium signaling to plant stress responses, particularly the induction of specific plant immunity against pathogens [69]. The importins belong to the karyopherin-alpha family involved in nuclear-cytoplasmic transport of macromolecules including hormones and phytoalexins decrease infection [70] (Chandra, 2012). The VSP is an endosomal polypeptides transporter necessary for protein translocation in the endoplasmic reticulum (ER) [71], and the Sec61 is an ER-localized protein that mediates the translocation of signal peptides. These proteins could be important for delivering defense-related protein complexes to the site of pathogenic invasion. Similarly, [72]. Based on these results we propose a model for the E14-Phy phenotype after PI inoculation (Fig. 9). In this model, the specific accumulation of defense proteins such as endochitinases, Ei1, HSPs, MAPKs, GSTs, WRKY transcription factor, BR, and Ca + signaling indicate E14-Phy deployed a strong specific immune response against PI, which underpinned the resistant phenotype observed in E14-Phy in contrast to FA-Phy.

### Summary

In this study, we applied ITRAQ-based quantitative proteomics to characterize the response of three Chinese potato cultivars: Favorita (FA), Mira (MA), and E-malingshu N0.14 (E14) infected with late blight disease pathogen *P. infestans*. After inoculation, phenotypic observations revealed that Favorita is susceptible, Mira is tolerant, and E-malingshu N0.14 is immune to late blight disease pathogen. The examination of protein abundance pattern identified potato proteins that were directly altered by the pathogen in the three cultivars, they include shared that were differentially regulated as well as cultivar specific proteins that were induced or repressed in response to PI. GO and KEGG analysis highlighted significant differences and similarities between these three cultivars in terms of defense response to the late blight disease pathogen. Specifically, we identified potato proteins that might be essential to establish tolerance and immunity against late blight disease pathogen *P. infestans*. For example, the repression of PR proteins, ABA, and shikimate pathway in FA-Phy boosted LBD susceptibility in FA plants. Whereas the induction of antimicrobial proteins, antioxidants, protease inhibitors, phenylpropanoid pathway, and repression of nutrient transport proteins enhanced PI tolerance in MA-Phy. Likewise, the induction of immune responses such as LRR receptor-like kinases, mitogen-activated protein kinase, serine/threonine kinases, enzyme regulators, WRKY transcription factors, terpenoid and polyketides, hormone signaling, and transport proteins specified immunity in E14-Phy. These proteins reported in this study are a valuable resource that can be used for developing tolerant and resistant varieties in potato breeding.

## Methods

### Plant materials and sample preparation

All the plant materials were originally possessed by Southern Potato Research Center of China, Enshi, Tujia and Miao Autonomous Prefecture, Hubei Province. The experimental research on the potato plants including sample collection was performed under institutional guidelines under the local legislation. The Favorita (FA), Mira (MA), and E-malingshu NO. 14 (E14) plants were sowed in a control room with growing conditions set at 20 °C, 16:8 light to dark cycle, and 70% relative humidity. At the 5-leaf stage, potato plants were divided into three groups of control plants (FA, MA, and E14) and three groups of FA, MA, and E14 plants inoculated with PI. Hereafter FA-Phy, MA-Phy, and E14-Phy. Each group contained six replicates. Inoculation was performed by spraying the whole plant with an encysted zoospore suspension from the same PI isolate until leaf surfaces were saturated with the zoospore suspension (15,000 sporangia/ml). The control groups were sprayed with water. To ensure pathogenic infection, the humidity was maintained at 100% for two days after inoculation and then adjusted to 90% for the rest of the experiment. For each cultivar, the control and the inoculated group samples were harvested in triplicates, 5 days after inoculation. For each replicate, three to four samples of fully expanded upper leaves were collected. All the materials were rapidly frozen in liquid nitrogen and stored at − 80 °C until use.

### Protein extraction, digestion, and iTRAQ labeling

Protein extraction was performed according to the method described by [73]. Briefly, all samples (FA, MA, and E14) and (FA-Phy, MA-Phy, and E14-Phy) were finely ground in liquid nitrogen, then the proteins were extracted following the procedure described by [36]. The samples are quickly transferred to a pre-chilled 1.5 mL centrifuge tube in liquid nitrogen, and 1 mL of pre-chilled 10% TCA / acetone (containing 65 mM DTT) was added to each sample and was kept at − 20 °C in the refrigerator for 2 h. Next, the samples were Centrifuged at 12000 g, 45 min, 4 °C, and the removed supernatant, then add − 20 °C precooled pure acetone, place in − 20 °C refrigerator for 2 h, afterward centrifuged at 12000 g, 45 min, 4 °C, remove supernatant, and this step was repeated three times. The obtained precipitate was lyophilized and about 800 mg of lyophilized powder was transferred to a 1.5 mL centrifuge tube and add 1000 μL of SDT protein lysate (4% SDS, 100 mM Tris-HCl, 100 mM DTT, PH8.0) was added, placed in a boiling water bath at 100 °C for 10 min, and ice bath for 10 min (35 W 2 s, interval 8S), and then placed in 100 °C boiling water bath for another 5 min. Finally, samples were centrifuged at 14000 g for 30 min, and the supernatant was collected and was filtered with a 0.22 μm ultrafiltration tube. We used 1 μL of the filtrate for quantification by BCA method, the remaining filtrate is stored at − 80 °C.

For each sample, 200 μg of protein extracts were denatured and reduced, and the cysteines blocked using iTRAQ reagents (8plex, AB Sciex, CA, USA), according to the manufacturer’s protocol. Next, proteins were diluted with five volumes of 50 mM TEAB to reduce urea concentration to 1.4 M, and twice digested with trypsin (Thermo Scientific, CA, USA) at a trypsin/protein ratio of 1:300 (37 °C, overnight. and then for 3 h). The resulting peptide solution was concentrated in a vacuum centrifuge and diluted with 70 μl of 100%ethanol. Afterward, the digested peptides were labeled with iTRAQ reagents. Samples were then mixed in equal ratios and dried in a vacuum centrifuge to remove the ethanol. Two 8-plex iTRAQ-labelled peptide mixtures were prepared. The first mixture contained proteins extracted from control samples (FA, MA, and E14) while the second mixture contained PI treated samples (FA-Phy, MA-Phy, and E14-Phy), including three biological replicates of isotopic labeling.

### Peptide fractionation with high-pH reversed-phase chromatography separation

Labeled and mixed peptides were subjected to High-pH Reversed-Phase Fractionation in the 1100 Series HPLC Value System (Agilent) equipped with a Gemini-NX (Phenomena, 00F-4453-E0) column (4.6 × 150 mm, 3 μm, 110 Å). Peptides were eluted at a flow rate of 0.8 mL/min. Buffer A consisted of 10 mM Ammonium acetate (pH 10.0) and buffer B consisted of 10 mM Ammonium acetate, 90% v/v CAN (pH 10.0). Buffer A and B were both filter-sterilized. The following gradient was applied to perform separation: 100% buffer A for 40 min, 0–5% buffer B for 3 min, 5–35% buffer B for 30 min, 35–70% buffer B for 10 min. Then, 70–75% buffer B for 10 min, 75–100% buffer B for 7 min, 100% buffer B for 15 min, and 100% buffer A for 15 min. The elution process was monitored by measuring absorbance at 214 nm, and fractions were collected every 75 s. Finally, the fractions collected were combined into 10 pools. Each fraction was concentrated via vacuum centrifugation and was reconstituted in 40 μL of 0.1% v/v trifluoroacetic acid. All samples were stored at − 80 °C until further analysis.

### LC-MS/MS analysis

The iTRAQ-labeled samples were analyzed using the Easy-nLC nanoflow HPLC system connected to Orbitrap Elite mass spectrometer (Thermo Fisher Scientific, San Jose, CA, USA). A total of 1 μg of each peptides sample was loaded onto the Thermo Scientific EASY column (two columns) using an autosampler at a flow rate of 150 nL/min. The sequential separation of peptides on Thermo Scientific EASY trap column (100 μm × 2 cm, 5 μm, 100 Å, C18) and analytical column (75 μm × 25 cm, 5 μm, 100 Å, C18) was achieved with a segmented 2 h gradient from Solvent A (0.1% formic acid in water) to 35% Solvent B (0.1% formic acid in 100% ACN) for 100 min, followed by 35–90% Solvent B for 12 min and then 90% Solvent B for 8 min. The mass spectrometer was operated in positive ion mode, and MS spectra were acquired over a range of 350–2000 m/z. Resolving powers of the MS scan and MS/MS at 100 m/z for the Orbitrap Elite were set as 60,000 and 15,000, respectively. The top sixteen most intense signals in acquired MS spectra were selected for further MS/MS analysis. The isolation window was 1 m/z, and ions were fragments through higher energy collisional dissociation with normalized collision energies of 35 eV. The maximum ion injection time was set at 50 ms for the survey scan, and 150 ms for the MS/MS scans, and the automatic gain control target values for full san modes were set to 10 × 10–6 and for MS/MS was 5 × 104. The dynamic exclusion duration was 30s.

### Protein identification and quantitation

MS/MS spectra were searched using the MASCOT engine (Matrix Science, London, UK; version 2.2) embedded into Proteome Discoverer 1.3 (Thermo Electron, San Jose, CA, USA) against UniProt plant database (134,648 sequences) and the decoy database. Search parameters include: monoisotopic mass; trypsin as cleavage enzyme; two max missed cleavages; iTRAQ 8 0plex (N-term), iTRAQ 8 plex (K) and carbamidomethylation of cysteine as fixed modifications; and oxidation of methionine as variable modifications (Additional file 9: Fig. S4). Peptide mass tolerance of ±20 ppm and fragment mass tolerance of 0.1 Da were used for parent and monoisotopic fragment ions, respectively. Results were filtered based on a false discovery rate of (FDR) ≤0.01. Relative quantitative analyses of proteins were based on ratios of iTRAQ reporter ions from all unique peptides representing each protein (Additional file 10: Fig. S5–8). For protein quantitation, each reporter ion channel was summed across all quantified proteins and normalized assuming equal protein loading of all ten samples. The protein ratios of each sample were normalized to the iTRAQ-126 label [74]. The mass spectrometry proteomics data have been deposited to the ProteomeXchange Consortium (http://proteomecentral.proteomexchange.org) via the iProX partner repository [75] with the dataset identifier PXD014647.

### Bioinformatics and statistical analysis

Proteins of *P*-values < 0.05 by Student T-test and a fold-change of > 1.20 in abundance between any two groups were considered significant. I.e., in a pairwise comparison between *P. infestans* treated and untreated control samples of each cultivar FA-Phy VS FA; MA-Phy VS MA and E14-Phy VS E14. Only proteins identified by two or more peptides with a *p*-value < 0.05, ≥ 1.2-fold change were classified as differentially abundant proteins. UniProt database (http://www.uniprot.org) [76] and Blast2GO (Version 2.7.2) [77] were used for GO terms classification of DAPs. Enriched GO terms were identified with Fisher’s Exact Test and hypergeometric distribution test cutoff of 0.05. Information on the biological pathways of the DAPs was obtained from the Kyoto Encyclopedia of Genes and Genomes pathways database [78]. Visualization of these pathways and enrichment analysis was performed using the KOBAS 2.0 software.

### Antibodies and Western-blot analyses

For western blot, the procedures of electrophoresis, transfer, and immunodetection were performed according to [79]. The primary antibodies used were as follows: antibody for the Serine/threonine-protein phosphatase (I6XKY2, PhytoAB PHY1724S, 1:1000); Protein CLP1 homolog (M1BQC2, PhytoAB PHY0964S, 1:1000); Probable glutathione S-transferase (P32111, PhytoAB PHY1514S, 1:1000); Putative endochitinase (Q2HPK8, PhytoAB PHY1514S, 1:1000). Horseradish peroxidase-conjugated anti-rabbit IgG (Bio-Rad, dilution 1: 5000) were used as secondary antibodies. After immunodetection, the intensity of the immunostained bands was normalized for the total protein intensities measured by Coomassie blue from the same blot [80]. The images were subjected to a densitometric analysis performed using Quantity One software (Bio-Rad).

### RNA extraction and quantitative real-time PCR (qPCR)

Total RNA was extracted from each sample using TRIZOL reagent (Invitrogen, Carlsbad, CA, USA). The RNA quantity and quality were determined with a NanoDrop 2000 spectrophotometer (Thermo, USA) according to the manufacturer’s instructions, after which cDNA was synthesized using the PrimeScript Reverse Transcriptase Kit (Takara, Dalian, China) for quantitative real-time polymerase chain reaction (qPCR). The gene-specific primers for the qPCR are listed in Additional file 2: Table S1. The PCR condition is as follows, 40 cycles of 95 °C for 15 s and 60 °C for 30 s). The gene expression levels were quantified relative to the potato *ef1a* gene [24] with 2–ΔΔCT method [81]. Each reaction was performed in three replicates. Primers used for qPCR are listed in Additional file 8: Table S5.

## Supplementary Information

**Additional file 1: Fig.S1.** Protein sequencing statistics. Blue bar represents MS spectrum, PSMs, Peptide, Unique peptide, and Protein in control (FA, MA, E14) and infected potato plants (FA-Phy, Ma-Phy, and E14-Phy).

**Additional file 2: Table S1.** Complete list of peptides and proteins identified in control (FA, MA, E14) and infected (FA-Phy, MA-Phy, and E14-Phy) potato plants. The list is organized by protein accession number.

**Additional file 3: Table S2.** Complete list of differentially abundant proteins between control (FA, MA, E14) and infected (FA-Phy, MA-Phy, and E14-Phy). The list is organized by protein accession number.

**Additional file 4: Fig. S2.** Pearson correlation analysis of biological replicates of control (FA, MA, E14) and infected potato plants (FA-Phy, Ma-Phy, and E14-Phy). The red box indicates a high positive correlation. The blue box indicates a low degree of correlation.

**Additional file 5: Table S3.** A list of overlapping proteins among the three comparisons in FA-Phy vs. FA, MA-Phy vs. MA, and E14-Phy vs. E14. The list is organized by protein accession number.

**Additional file 6: Fig. S3.** GO enrichment of biological processes categories. A, B. Up-regulated, and down-regulated DAPs of FA-Phy. C, D. Up-regulated, and down-regulated DAPs enriched in MA-Phy. E, F. Up-regulated and down-regulated DAPs enriched in E14-Phy.

**Additional file 7: Table S4.** A list of proteins associated with KEGG pathways in FA-Phy vs. FA, MA-Phy vs. MA, and E14-Phy vs. E14. The list is organized by protein accession number.

**Additional file 8: Table S5.** The list of gene-specific primers selected for qRT-PCR analysis. The list is organized by protein accession number.

**Additional file 9: Figure S4.**

**Additional file 10: Figure S5-8.**

## Data Availability

The mass spectrometry proteomics data have been deposited to the ProteomeXchange Consortium (http://proteomecentral.proteomexchange.org) via the iProX partner repository with the dataset identifier PXD014647.

## Abbreviations

LBD: Late blight disease
PI: *Phytophthora infestans*
FA: Favorita
MA: Mira
E14: E-malingshu N0.14
R: Resistance
PAMPs: Pathogen-associated molecular patterns
PRRs: Pattern-recognition receptors
PTI: PAMP-triggered immunity
ETS: Effector-triggered susceptibility
NBS: N-terminal nucleotide-binding site
LRR: C-terminal leucine-rich repeat
PCD: Programmed cell death
ITRAQ: Isobaric tags for relative and absolute quantification
TMT: Tandem mass tags
DAPs: Differentially abundant proteins
GO: Gene ontology
qRT-PCR: Quantitative real-time polymerase chain reaction
cDNA: complementary deoxyribonucleic acid
RNA: Ribonucleic acid
LC-MS/MS: Liquid chromatography-mass spectrometry
