# Supplementary figures and images for: Comparative proteomics of three Chinese potato cultivars to improve understanding of potato molecular response to late blight disease
Source: BMC Genomics. 2020 Dec 9;21:880. doi: 10.1186/s12864-020-07286-3 (PMC7727141; doi:10.1186/s12864-020-07286-3)

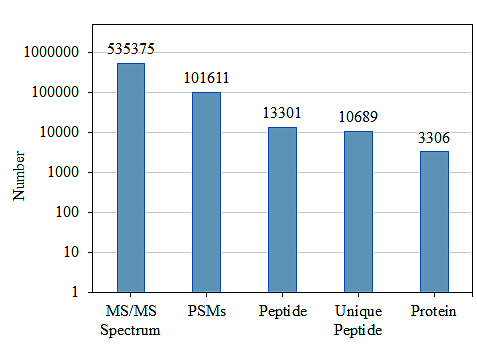

Supplement: Supplementary file 1 — Additional file 1: Fig.S1. Protein sequencing statistics. Blue bar represents MS spectrum, PSMs, Peptide, Unique peptide, and Protein in control (FA, MA, E14) and infected potato plants (FA-Phy, Ma-Phy, and E14-Phy). [file 12864_2020_7286_MOESM1_ESM.tiff]

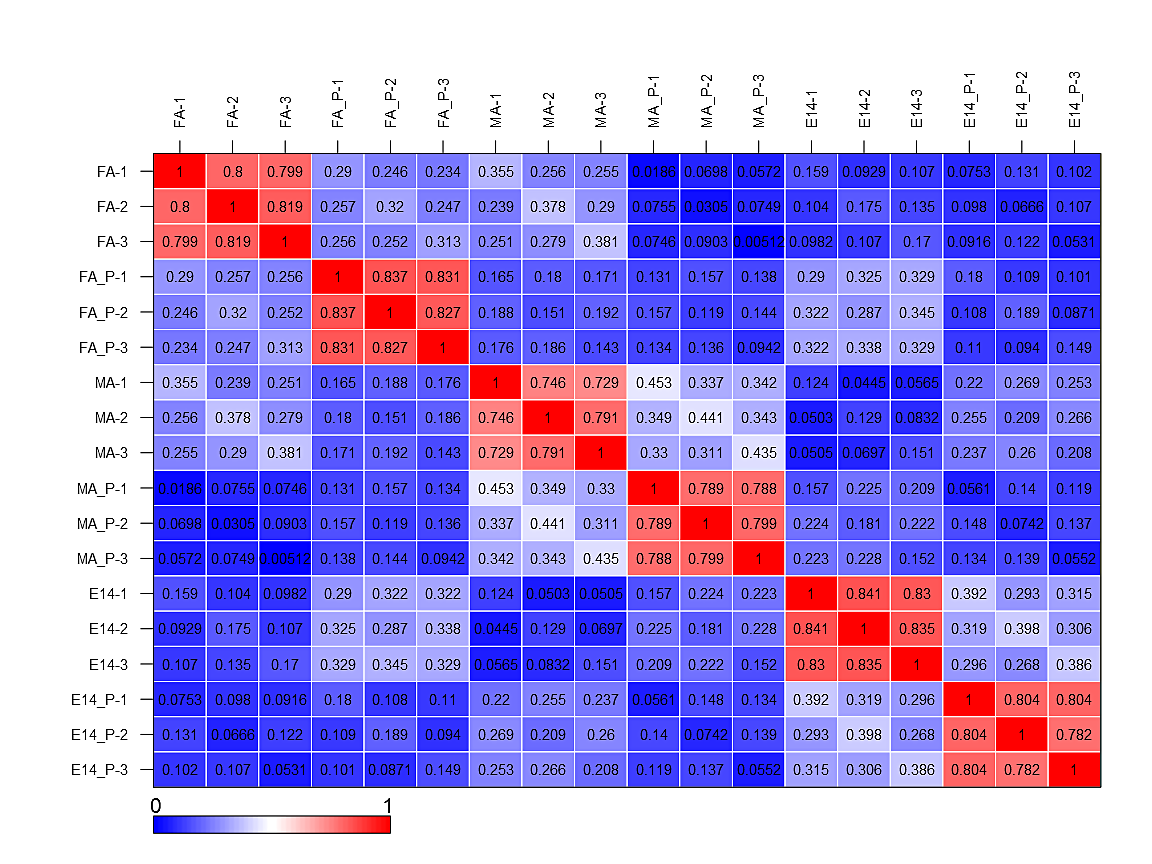

Supplement: Supplementary file 4 — Additional file 4: Fig. S2. Pearson correlation analysis of biological replicates of control (FA, MA, E14) and infected potato plants (FA-Phy, Ma-Phy, and E14-Phy). The red box indicates a high positive correlation. The blue box indicates a low degree of correlation. [file 12864_2020_7286_MOESM4_ESM.tiff]

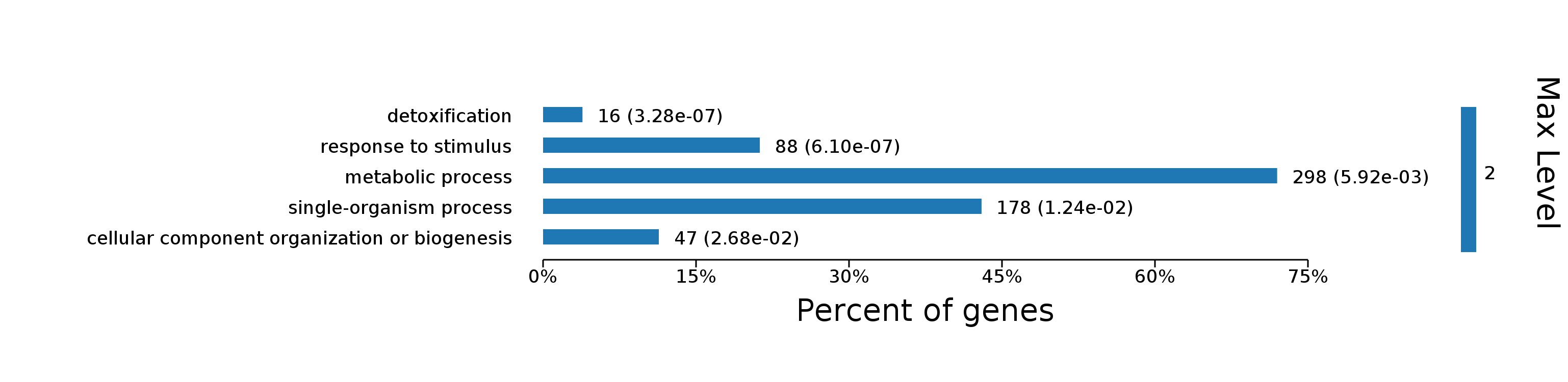

Supplement: Supplementary file 6 — Additional file 6: Fig. S3. GO enrichment of biological processes categories. A, B. Up-regulated, and down-regulated DAPs of FA-Phy. C, D. Up-regulated, and down-regulated DAPs enriched in MA-Phy. E, F. Up-regulated and down-regulated DAPs enriched in E14-Phy. [file 12864_2020_7286_MOESM6_ESM.zip › Fig S3A (1).tiff]

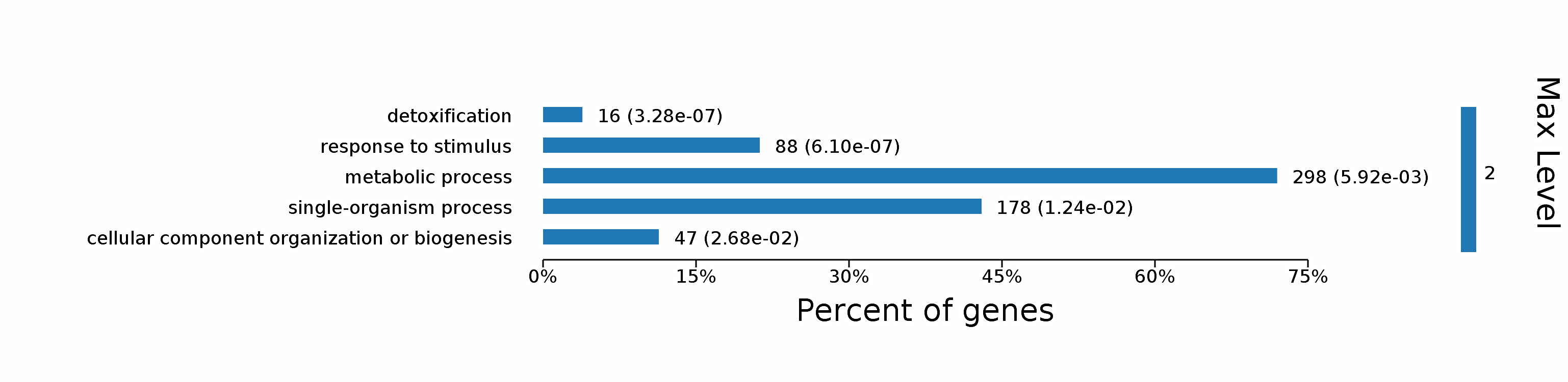

Supplement: Supplementary file 6 — Additional file 6: Fig. S3. GO enrichment of biological processes categories. A, B. Up-regulated, and down-regulated DAPs of FA-Phy. C, D. Up-regulated, and down-regulated DAPs enriched in MA-Phy. E, F. Up-regulated and down-regulated DAPs enriched in E14-Phy. [file 12864_2020_7286_MOESM6_ESM.zip › Fig S3A.tiff]

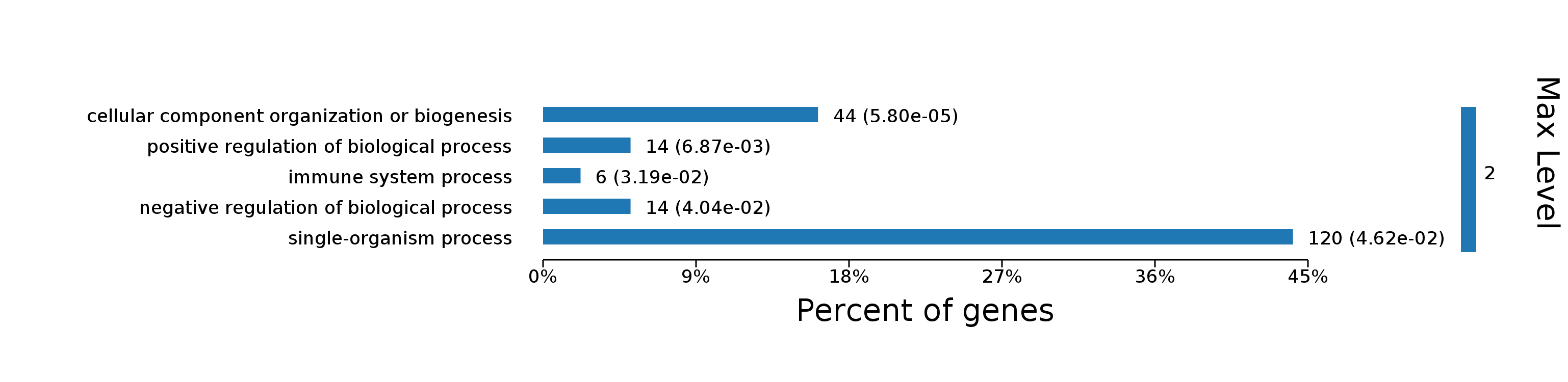

Supplement: Supplementary file 6 — Additional file 6: Fig. S3. GO enrichment of biological processes categories. A, B. Up-regulated, and down-regulated DAPs of FA-Phy. C, D. Up-regulated, and down-regulated DAPs enriched in MA-Phy. E, F. Up-regulated and down-regulated DAPs enriched in E14-Phy. [file 12864_2020_7286_MOESM6_ESM.zip › Fig S3B (1).tiff]

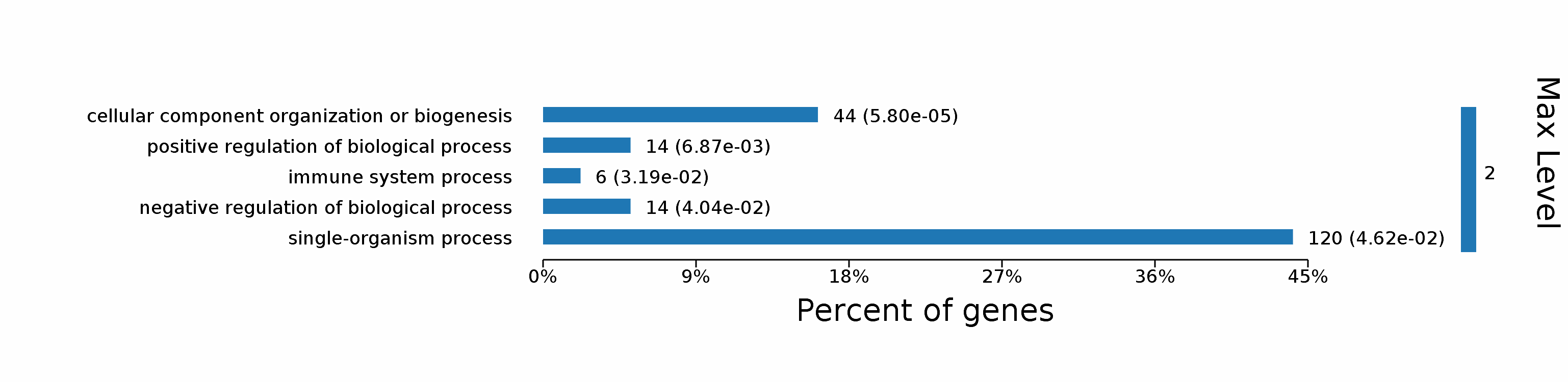

Supplement: Supplementary file 6 — Additional file 6: Fig. S3. GO enrichment of biological processes categories. A, B. Up-regulated, and down-regulated DAPs of FA-Phy. C, D. Up-regulated, and down-regulated DAPs enriched in MA-Phy. E, F. Up-regulated and down-regulated DAPs enriched in E14-Phy. [file 12864_2020_7286_MOESM6_ESM.zip › Fig S3B.tiff]

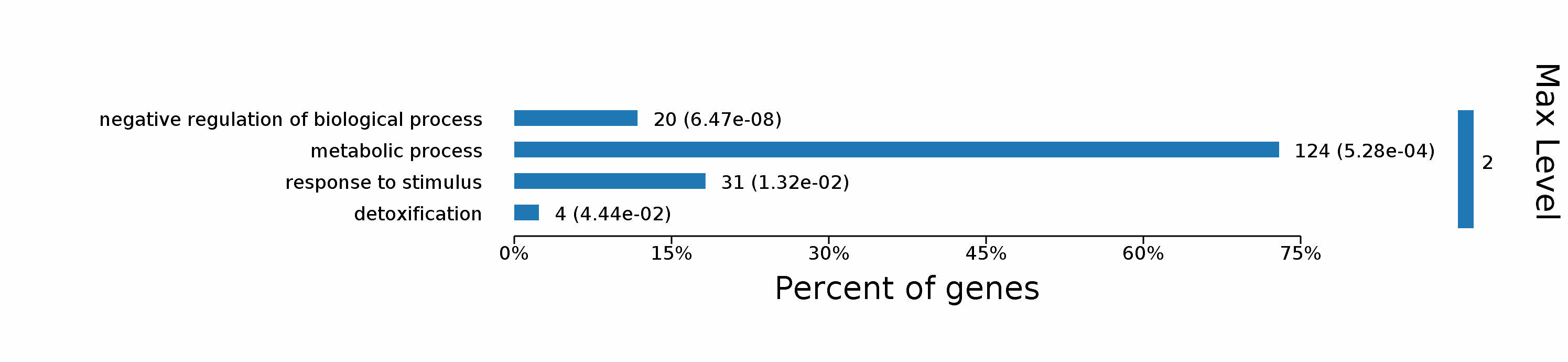

Supplement: Supplementary file 6 — Additional file 6: Fig. S3. GO enrichment of biological processes categories. A, B. Up-regulated, and down-regulated DAPs of FA-Phy. C, D. Up-regulated, and down-regulated DAPs enriched in MA-Phy. E, F. Up-regulated and down-regulated DAPs enriched in E14-Phy. [file 12864_2020_7286_MOESM6_ESM.zip › Fig S3C.tiff]

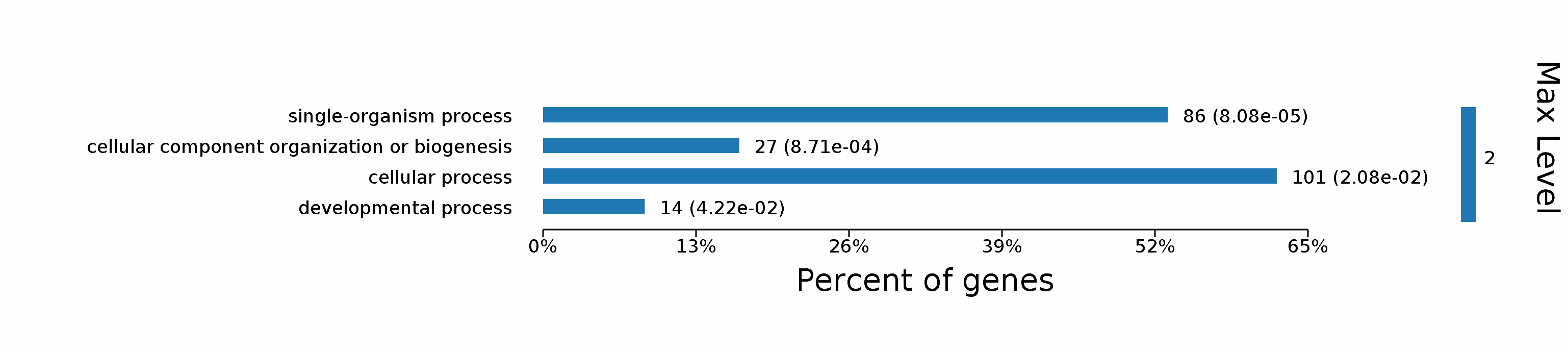

Supplement: Supplementary file 6 — Additional file 6: Fig. S3. GO enrichment of biological processes categories. A, B. Up-regulated, and down-regulated DAPs of FA-Phy. C, D. Up-regulated, and down-regulated DAPs enriched in MA-Phy. E, F. Up-regulated and down-regulated DAPs enriched in E14-Phy. [file 12864_2020_7286_MOESM6_ESM.zip › Fig S3D.tiff]

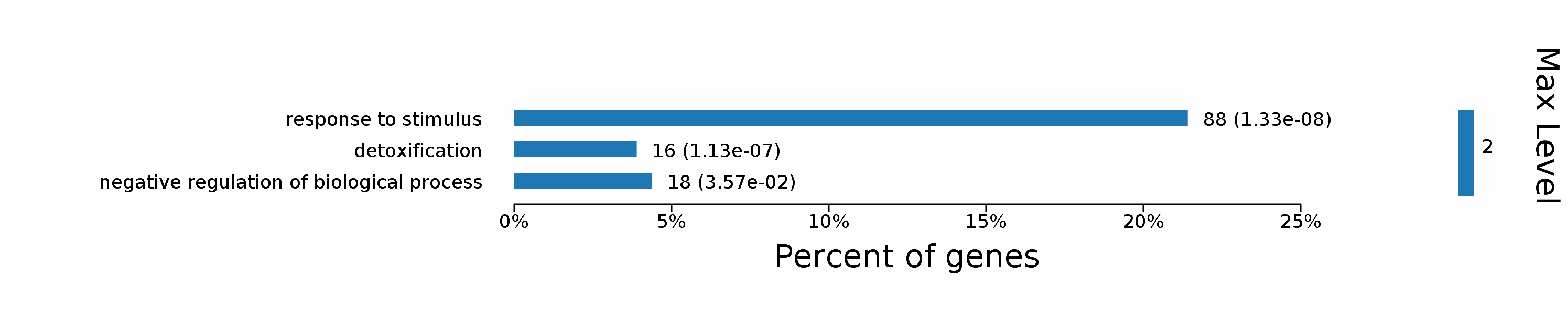

Supplement: Supplementary file 6 — Additional file 6: Fig. S3. GO enrichment of biological processes categories. A, B. Up-regulated, and down-regulated DAPs of FA-Phy. C, D. Up-regulated, and down-regulated DAPs enriched in MA-Phy. E, F. Up-regulated and down-regulated DAPs enriched in E14-Phy. [file 12864_2020_7286_MOESM6_ESM.zip › Fig S3E.tiff]

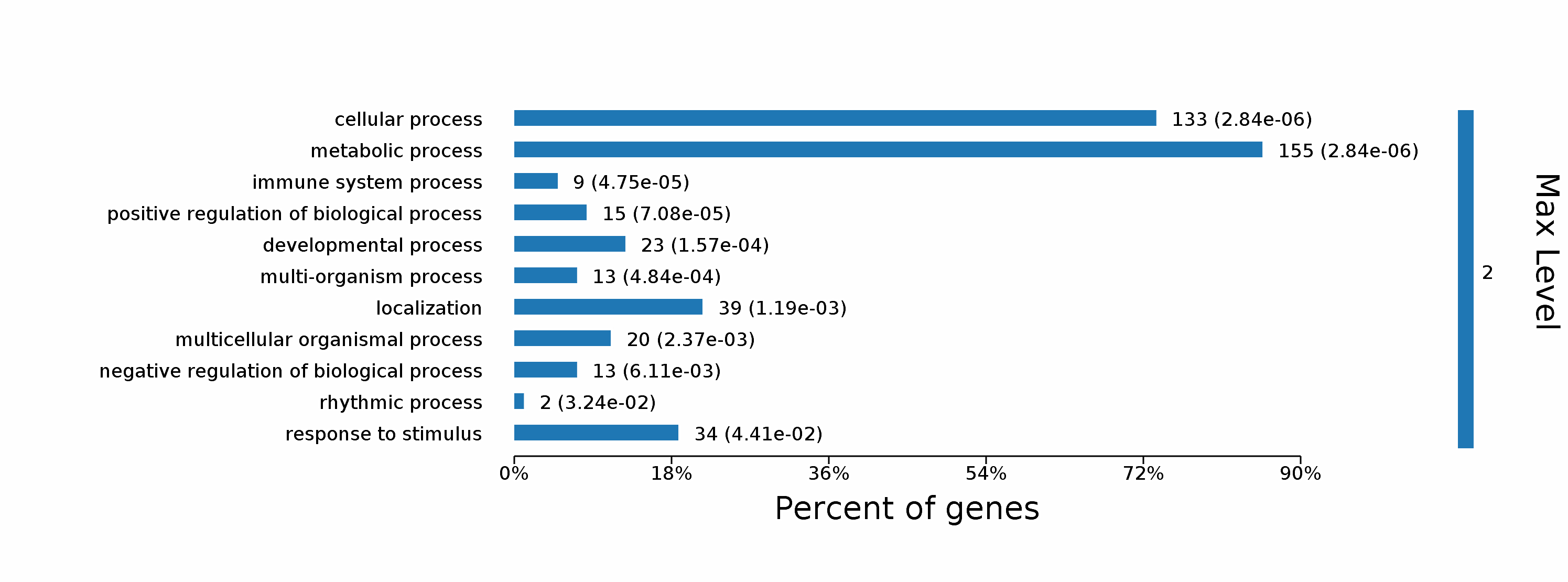

Supplement: Supplementary file 6 — Additional file 6: Fig. S3. GO enrichment of biological processes categories. A, B. Up-regulated, and down-regulated DAPs of FA-Phy. C, D. Up-regulated, and down-regulated DAPs enriched in MA-Phy. E, F. Up-regulated and down-regulated DAPs enriched in E14-Phy. [file 12864_2020_7286_MOESM6_ESM.zip › Fig S3F.tiff]

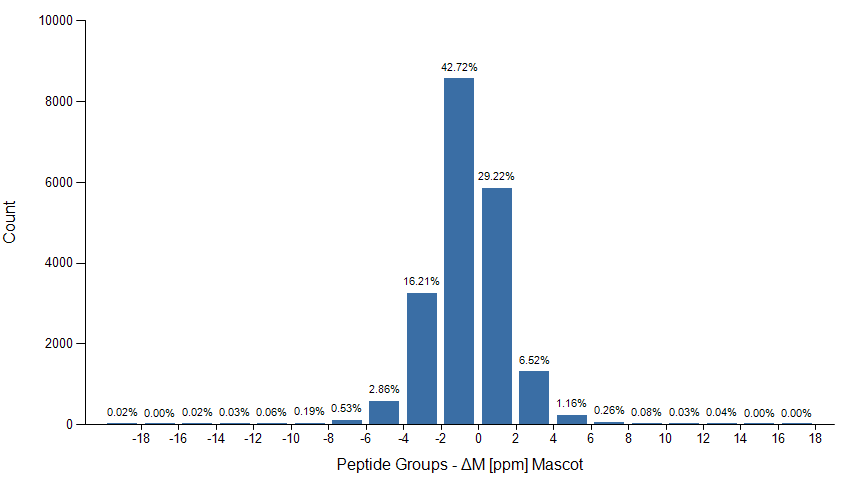

Supplement: Supplementary file 9 — Additional file 9: Figure S4. [file 12864_2020_7286_MOESM9_ESM.png]

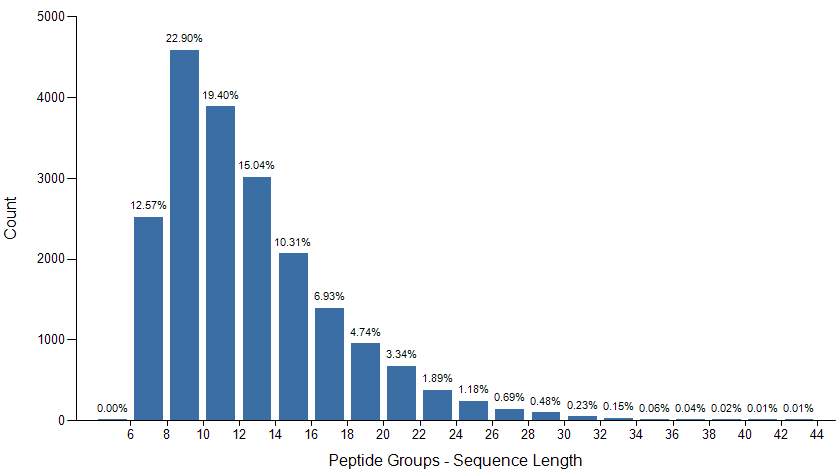

Supplement: Supplementary file 10 — Additional file 10: Figure S5-8. [file 12864_2020_7286_MOESM10_ESM.zip › Fig S5.png]

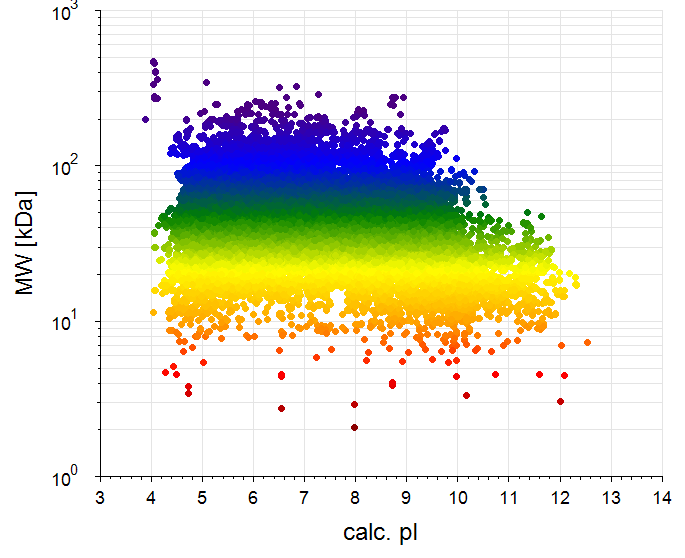

Supplement: Supplementary file 10 — Additional file 10: Figure S5-8. [file 12864_2020_7286_MOESM10_ESM.zip › Fig S6.png]

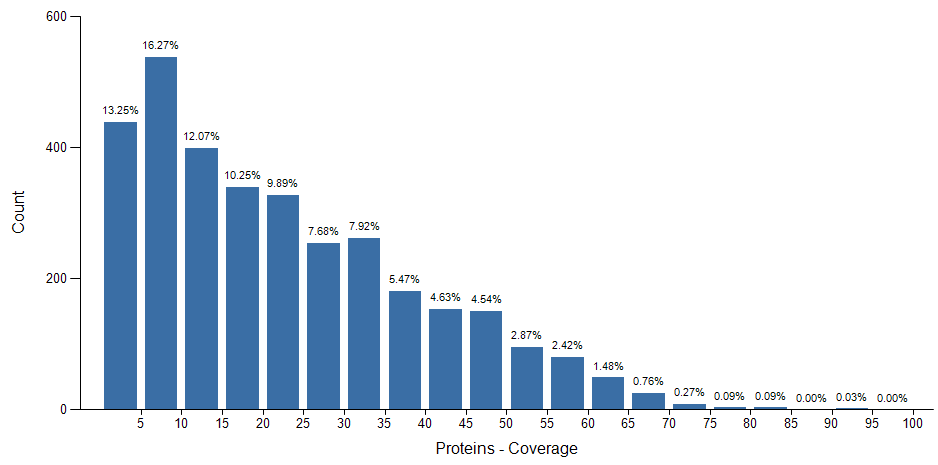

Supplement: Supplementary file 10 — Additional file 10: Figure S5-8. [file 12864_2020_7286_MOESM10_ESM.zip › Fig S7.png]

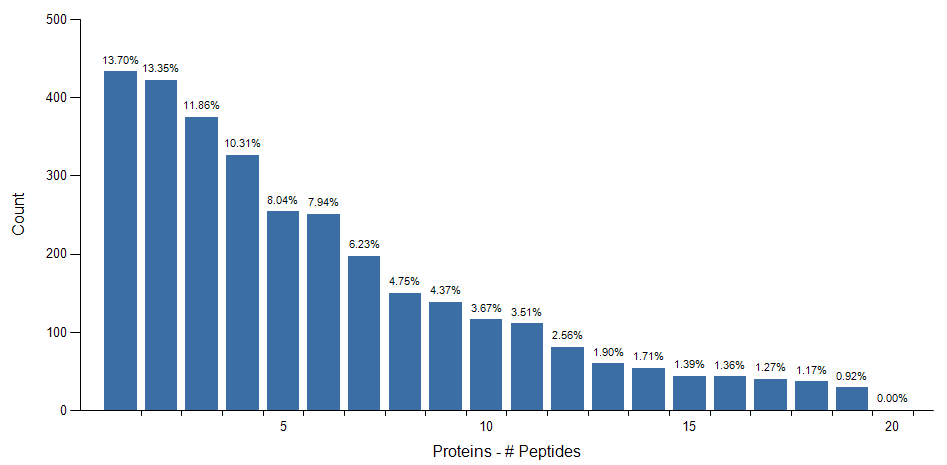

Supplement: Supplementary file 10 — Additional file 10: Figure S5-8. [file 12864_2020_7286_MOESM10_ESM.zip › Fig S8.png]
